# Regularized ensemble Kalman inversion for robust and efficient gravity data modeling to identify mineral and ore deposits

**DOI:** 10.1038/s41598-025-30141-y

**Published:** 2025-11-27

**Authors:** Dharma Arung Laby, S. Sungkono, Arkoprovo Biswas

**Affiliations:** 1 Department of Geophysical Engineering, Faculty of Civil, Planning, and Geoengineering, Institut Teknologi Sepuluh Nopember, Surabaya, 60111 Indonesia; 2 Department of Physics, Faculty of Science and Data Analytics, Institut Teknologi Sepuluh Nopember, Surabaya, 60111 Indonesia; 3 Geological Studies Unit, Physics and Earth Science Division, Indian Statistical Institute, Kolkata, 700108 West Bengal India

**Keywords:** Gravity, Ensemble kalman inversion, Tikhonov regularization, Ore deposit, Mineral exploration, Geology, Geophysics

## Abstract

Modeling mineral and ore bodies from gravity anomalies remains challenging in geophysical exploration due to the ill-posed and non-unique nature of the inverse problem, particularly under conditions of noisy or sparse data. Established inversion methods, including local optimization and metaheuristic algorithms, often require extensive parameter tuning and may yield unstable or poorly constrained solutions. This study proposes a regularized ensemble Kalman inversion (EKI) framework enhanced by Tikhonov regularization to improve numerical stability and mitigate sensitivity to ensemble degeneracy, thereby enabling efficient uncertainty quantification through ensemble statistics. Controlled numerical experiments show that the ensemble size is larger than $\:100$ with moderate regularization, we can achieve an optimal balance between convergence stability and model resolution. Benchmarking against established metaheuristic algorithms (PSO, VFSA, and BA) suggests superior computational efficiency and stable convergence. Synthetic and real gravity data inversion (chromite, Pb-Zn, sulphide, and Cu-Au deposits) suggests that the regularized EKI yields stable, geologically consistent results with prior interpretations and drilling data. These results highlight the regularized EKI framework as a robust and efficient tool for mitigating mining risks and supporting strategic decision-making in mineral exploration.

## Introduction

Historically, the gravity method has served as a fundamental tool in geophysical prospecting for mineral and ore exploration because of its ability to detect subsurface density variations associated with ore deposits^1,2^. By measuring the gravity field at multiple surface points, this method effectively identifies and maps gravity anomalies caused by mineralized bodies^3–5^. Moreover, gravity data can be directly used to calculate ore reserves^2^. Numerous studies have demonstrated its successful application in exploring various mineral resources, such as sulphides^6^, manganese^7^, zinc^8^, chromite^9^, tin^10^, uranium^11^, iron^12^, and copper-gold deposits^4^. Gravity data are considered economically significant for mineral exploration. Therefore, modeling mineral or ore bodies from gravity measurements is critical for reducing mining risks and supporting strategic investment planning^5,11^.

Modeling mineral or ore bodies using idealized geometric models (e.g., spheres, horizontal and vertical cylinders) remains widely adopted and continues to attract significant attention^5,13–15^. These models are especially suitable for geological settings with distinct anomalies that can be interpreted as isolated bodies, such as individual ore deposits. The modeling process involves inverting measured gravity data to estimate source parameters (e.g., depth, amplitude coefficient, body location, and shape factors) associated with the idealized models^15,17,18^. Hence, inversion plays a vital role in characterizing the source geometry of gravity anomalies.

Various inversion algorithms, including local and global search methods, have been proposed to estimate these parameters. Local methods, such as least-squares inversion and its variants, have been extensively studied^13,19–24^. These techniques typically estimate a limited set of parameters, such as depth, shape factor, and amplitude coefficient. However, accurate inversion requires simultaneous estimation of all model parameters, increasing ambiguity due to interdependencies^16,25^. Despite advancements in numerical techniques, the non-uniqueness and resolution of gravity data inversion remain significant challenges^18,27^. As a derivative-based method, least-squares inversion is sensitive to the initial guess. It often fails to thoroughly explore the parameter space, leading to poor solutions, particularly in ill-posed inverse problems^3^.

Global optimization methods, particularly metaheuristic algorithms, have been developed to address these issues. These algorithms can avoid local minima and explore the solution space more comprehensively than the existing algorithms^27^. Prominent examples include very fast simulated annealing (VFSA)^16,17^, differential evolution (DE)^7^, particle swarm optimization (PSO)^28,29^, the bat algorithm (BA)^3^, and the dual classification learning Rao (DL Rao) algorithm^5^. These methods have demonstrated promising results in addressing the non-uniqueness of gravity inversion by effectively balancing exploration and exploitation. However, many such systems require extensive parameter tuning, which increases the implementation complexity and computational cost. Improper parameter selection can result in premature convergence and inaccurate subsurface models.

Therefore, alternative inversion strategies that require minimal parameter tuning and efficient uncertainty handling are needed. Compared to metaheuristics, data assimilation techniques provide a systematic framework for integrating observational data with forward models, enabling more robust and efficient parameter estimation^30,31^. Among these methods, ensemble Kalman inversion (EKI) has emerged as a powerful and computationally attractive geophysical inversion method. The proposed method offers derivative-free estimation, straightforward implementation, and built-in uncertainty quantification, making it well-suited for complex, ill-posed inverse problems.

Ensemble Kalman inversion (EKI) is an advanced statistical approach in data assimilation^32^, which has been widely recognized as an effective method for solving Bayesian inverse problems^33^. Initially, EKI was developed for applications in weather forecasting^34,35^ and oceanography^36^. Since then, EKI has been successfully applied to various geophysical applications, including climate modeling^37^, reservoir characterization^38,39^, seismic tomography^40^, induced polarization and DC-resistivity^41,42^, and self-potential data analysis^43^. EKI is preferred for solving geophysical inverse problems due to its derivative-free nature and powerful computational capabilities^44^. EKI provides an efficient inversion approach, easy implementation, and uncertainty quantification without extensive parameter tuning^41–43^. EKI operates by leveraging an ensemble of parameter realizations to approximate the posterior distribution, combining the benefits of Monte Carlo sampling with the computational efficiency of Kalman filtering^45–47^. Despite its advantages, EKI is not without limitations. Numerical instability can hinder its performance, particularly when the covariance matrix of the model predictions becomes ill-conditioned or singular. This instability often leads to unreliable results or algorithm failure, particularly in noisy or incomplete data scenarios.

To address these limitations, this study introduces a regularized version of EKI that incorporates Tikhonov regularization into the Kalman gain computation. This enhancement stabilizes the inversion process by improving the covariance matrix conditioning, ensuring reliable updates even under noise or limited ensemble diversity^48–51^. This approach preserves EKI’s computational efficiency and uncertainty quantification while significantly enhancing its robustness and reliability. The proposed framework is capable of estimating model parameters, including depth $\:\left({z}\right)$, location of the source origin $\:\left({{x}}_{{0}}\right)$, amplitude coefficient $\:\left({A}\right)$, and shape factors $\left( {{{q}}\;{{and}}\;\mu } \right)$, along with their associated uncertainties derived from the posterior ensemble distribution. A key contribution of this study is the integration of Tikhonov regularization to address ill-posed problems and enhance the stability of EKI in gravity data inversion, reducing its sensitivity to noise and numerical precision errors.

The present work is organized as follows: section “Ensemble Kalman inversion” presents the development and principles of the EKI framework. Section “Methodology” describes the methodology, including forward gravity data modeling and EKI-based inversion. Section “Synthetic data inversion” discusses pre-inversion evaluation, followed by the results of synthetic-data inversion, including benchmarking. Section “Inversion of field data examples” presents field case studies from real gravity datasets. Finally, section “Conclusions” concludes with a summary of the findings and implications.

## Ensemble Kalman inversion

The Kalman filter (KF) was initially introduced by Kalman^52^ for recursive state estimation in Gaussian systems and later extended to nonlinear systems. However, the extended KF faces limitations due to the computational burden of covariance estimation in high-dimensional systems. To address this limitation, the ensemble Kalman Filter (EnKF) was introduced^36,53^. EnKF is a Monte Carlo-based approach that uses an ensemble of particles to estimate statistical properties, such as the covariance and mean of the model parameters^54^. Due to its effectiveness, EnKF has gained significant interest across various disciplines, including inverse problems^44^.

The ensemble Kalman inversion (EKI) framework is an adaptation of the ensemble Kalman filter (EnKF) framework for inverse problems^45^. While EnKF estimates system states by sequentially assimilating new data, EKI adapts this approach to iteratively refine unknown parameters. Each iteration updates the parameter estimates, resembling a pseudo-time evolution. Compared to EnKF, which continuously updates state variables with each new observation, EKI uses the entire dataset in a static setting to iteratively minimize the misfit between calculated and observed data, refining the parameters^33,45,47^. This makes EKI particularly useful for inverse problems, offering efficient parameter estimation, uncertainty quantification, and robustness to noisy data without requiring explicit derivatives^44,45,55^.

The goal of EKI is to estimate the unknown parameters *m* from the given observational data ${{{d}}_{{{obs}}}}$ expressed as

1 $${d_{obs}}=\mathcal{G}\left( m \right)+~\eta$$

where $\mathcal{G}\left( m \right)$ denotes a forward modeling, and $\eta$ represents the noise of the observed data. The probabilistic distribution ${{P}}\left( {{{m|}}{{{d}}_{{{obs}}}}} \right)$ represents the solution of the inverse problem. This distribution expresses the probability of the model parameter *m* given the observed data ${{{d}}_{{{obs}}}}$. The proposed approach follows Bayes’ theorem as follows:

2 $$P\left( {m{{|}}{d_{obs}}} \right) \propto P\left( m \right)P\left( {{d_{obs}}{{|}}m} \right)$$

$P\left( m \right)$ represents the prior distribution of the model parameters, while $P\left( {{d_{obs}}{{|}}m} \right)$ denotes the likelihood. The expression $P\left( {m{{|}}{d_{obs}}} \right)$ defines the posterior distribution of the parameter. Direct sampling of the posterior distribution typically relies on Monte Carlo methods, which require millions of forward model evaluations^43,56^. To improve efficiency, global optimization methods with thresholding have been applied to estimate the model parameter *m* more effectively^57^.

The EKI process typically involves several steps : (1) ensemble initialization; (2) forward model evaluation; (3) ensemble statistical parameter calculation; (4) Kalman gain calculation; (5) parameter update; (6) convergence assessment^53,58^. Each of these steps is described in detail below.

1. *Ensemble initialization*: The process begins by generating an ensemble of ${N_e}$ parameter realizations, denoted as ${{M=\{ }}{{{m}}_{{i}}}{{\} }}_{{{{i=1}}}}^{{{{{N}}_{{e}}}}}$, sampled from an initial prior distribution. The ensemble members are drawn uniformly within parameter bounds:
  3 $$m_{i}^{{\left( 0 \right)}}\sim \mathcal{U}\left( {{M_{min}},{M_{max}}} \right)$$
  where $m_{i}^{{\left( 0 \right)}}$ represents the initial parameter set of the *i-*th ensemble member, and $\mathcal{U}\left( \cdot \right)$ denotes a uniform distribution within the parameter range.
2. *Forward model evaluation*: Each ensemble member is used as input to the forward model $\mathcal{G}$, which predicts the corresponding calculated data ${d_{cal,i}}$ :
  4 $$d_{{cal,i}} = {\mathcal{G}}\left( {m_{i} } \right)$$
  where $\mathcal{G}$ represent a geophysical model that relates the parameter ${m_i}$ to the observed data. The predicted values ${d_{cal,i}}$ are then compared with the observed data ${d_{obs}}$.
3. *Calculation of ensemble statistical parameters*: The statistical properties of the ensemble are computed in each iteration. The ensemble means of the parameters and predicted observations are calculated as
  5 $$\bar{m} = \frac{1}{{N_{e} }}\mathop \sum \limits_{{i = 1}}^{{N_{e} }} m_{i}$$
  6 $$\bar{d}_{{cal}} = \frac{1}{{N_{e} }}\mathop \sum \limits_{{i = 1}}^{{N_{e} }} d_{{cal,i}}$$
  The sample covariance matrices for parameter-data relationships are then estimated as follows:
  7 $$C_{{md}} = \frac{1}{{N_{e} - 1}}\mathop \sum \limits_{{i = 1}}^{{N_{e} }} \left( {m_{i} - \bar{m}} \right)\left( {d_{{cal,i}} - \bar{d}_{{cal}} } \right)^{T}$$
  8 $$C_{{dd}} = \frac{1}{{N_{e} - 1}}\mathop \sum \limits_{{i = 1}}^{{N_{e} }} \left( {d_{{cal,i}} - \bar{d}_{{cal}} } \right)\left( {d_{{cal,i}} - \bar{d}_{{cal}} } \right)^{T}$$
  where ${C_{md}}$ represents the cross-covariance between parameters *M* and observations, and ${C_{dd}}$ is the auto-covariance of the predictions.
4. *Kalman gain calculation*: To update parameters using the observed data, the Kalman gain *K* should be computed first. The Kalman gain *K* is a matrix that optimally weights the difference between observed data and model predictions to update parameter estimates in Kalman methods, minimizing posterior uncertainty^45,52,54^. It is generally calculated as follows^45,53^:
  9 $${{K=~}}{{{C}}_{{{md}}}}\left( {{{{C}}_{{{dd}}}}{{+}}{{{C}}_{{d}}}} \right){~^{{{-1}}}}$$
  where ${C_d}$ represents the covariance of the observational noise. This matrix is typically diagonal, with each diagonal element representing the observation noise variance at a given data point. The accuracy of the Kalman gain critically depends on the auto-covariance of the model parameters, which is approximated using a finite ensemble and may lead to unreliable updates^59^. Furthermore, if the parameter ensemble exhibits low variability and ${C_d}$ is undersized, the Kalman gain may become ill-conditioned or singular, leading to algorithm failure^43^. To handle this, Tikhonov regularization was implemented to stabilize Kalman gain computation. Regularization helps mitigate the effects of an ill-conditioned covariance matrix by adding a small positive term to the denominator in Eq. (9), thereby ensuring numerical stability and preventing singularities. The modified Kalman gain incorporating Tikhonov regularization is expressed as follows:
  10 $$K = C_{{md}} \left( {C_{{dd}} + C_{d} + \lambda I} \right)^{{~ - 1}}$$
  where $\lambda$ is the regularization parameter, and *I* is the identity matrix. The inclusion of $\lambda I$ serves to improve the conditioning of the inverse computation, enabling stable and reliable parameter updates while maintaining consistency with the observed data. The additional term improves the conditioning of matrix inversion, effectively damping unstable directions in the parameter space. Intuitively, the Tikhonov regularization acts as a smoothing mechanism, limiting the influence of ensemble noise and poorly constrained directions. This is particularly important for gravity inversion problems, which are often ill-posed and sensitive to shape-related parameters. By introducing a controlled level of bias, regularization enhances the stability and robustness of the inversion, ensuring reliable updates even in the presence of data noise or limited ensemble size.
5. *Parameter update*: Each ensemble member is updated using the computed Kalman gain and perturbed observations as follows:
  11 $${{m}}_{{{i}}}^{{{{k+1}}}}{{=m}}_{{{i}}}^{{{k}}}{{+K}}\left( {{{{d}}_{{{obs}}}}{{+}}{{{\varvec{\upeta}}}_{{i}}}{{-d}}_{{{{cal,i}}}}^{{{k}}}} \right)$$
  where ${\eta _i}\sim \mathcal{N}\left( {0,{C_d}} \right)$ represents synthetic noise added to the observations to account for uncertainty.
6. *Convergence assessment*: The iterative process continues until the misfit between predicted and observed data is minimized, which can be evaluated using an error metric, such as the root mean squared error (RMSE). Convergence is achieved when the error falls below a predefined threshold or when successive iterations exhibit minimal improvement. The final parameter estimates are obtained from the best-performing ensemble member.

In addition to using Tikhonov regularization to enhance the stability and performance of EKI, other strategies, such as greedy selection and boundary handling, have been implemented to maintain parameter feasibility and improve the inversion results^43,59^. Greedy selection ensures that only the best-performing ensemble members are retained, effectively refining the ensemble over successive iterations. Meanwhile, boundary handling techniques prevent parameters from assuming values beyond the physical limits defined by prior knowledge, thereby avoiding unrealistic solutions during the inversion process. These enhancements improve the robustness and reliability of the EKI framework for geophysical inverse problems, especially gravity data inversion.

## Methodology

This section describes the formulation of the gravity forward modeling used to calculate the gravity response from the given parameters, as well as the gravity data inversion procedure, utilizing the ensemble Kalman inversion (EKI) algorithm.

### Gravity data forward modeling

**Fig. 1:**
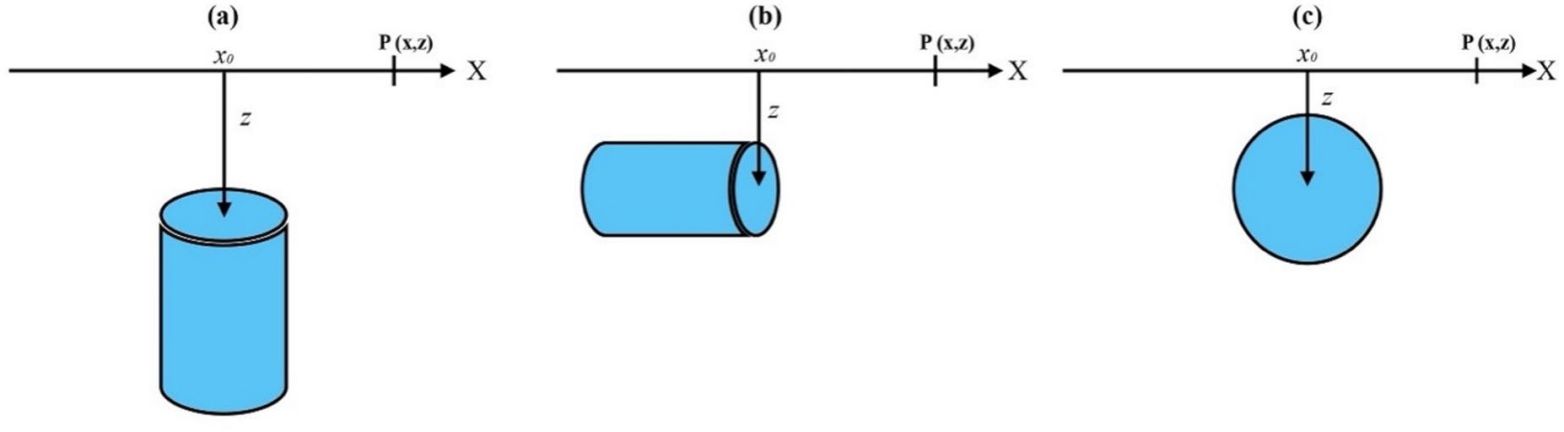
Illustration of different geometric models of causative bodies: (a) vertical cylinder; (b) horizontal cylinder; (c) sphere.

At a given location point ${x_i}$, the gravity anomaly along a profile is defined as

12 $${{g}}\left( {{{{x}}_{{i}}}} \right){{=}}\frac{{{{Az}}{{{~}}^{{\varvec{\upmu}}}}}}{{{{\left( {{{\left( {{{{x}}_{{i}}}~{{-~}}{{{x}}_{{0}}}} \right)}^{{{~2~}}}}{{+~z}}{{{~}}^{{2}}}} \right)}^{{{~q}}}}}}~~,i=1,2,~ \ldots ,~n$$

where ${x_0}$ represents the location of model’s origin, and *z* denotes the source depth. The shape factors *q* and $\mu$ influence the amplitude coefficient *A*, ensuring that the gravity anomaly *g* is expressed in mGal units. The definitions of *A*,* q*, and $\mu$ for different geometric models are provided in Table 1 and illustrated in Fig. 1.

**Table 1:** Definitions of *A*,* q*, and $\mu$ for different geometric models, where represents the density contrast (*g/cc*), $\gamma \sigma$ denotes the universal gravitational constant ($6.67384{{x}}{10^{ - 11}}~{{{m}}^{ - 3}}{{~k}}{{{g}}^{ - 1}}{{~}}{{{s}}^{ - 2}}$), and *r* corresponds to the radius of the models.

Geometry Body	A	$\varvec{\mu}$	q
Vertical cylinder	${{\varvec{\uppi}\varvec{\upgamma}\varvec{\upsigma}}}{r^2}$	0	0.5
Horizontal cylinder	$2\pi \gamma \sigma {r^2}$	1	1
Sphere	$\frac{4}{3}\pi \gamma \sigma {r^2}$	1	1.5

### Gravity data inversion using EKI

As described in the previous section, a gravity data inversion program using EKI was developed to estimate the model parameters of the source gravity anomalies. The EKI program was extended to improve its performance with additional features, including Tikhonov regularization for stability, greedy selection for optimal ensemble refinement, and boundary handling to maintain parameter feasibility.

The inversion program begins by initializing an ensemble of model parameters. First, the number of ensemble members (*N*_*e*_), the total number of iterations (${N_{iter}}$), and the observation noise covariance ${C_d}$ are defined. The choice of *N*_*e*_ influences the statistical representation of the parameter space: a higher *N*_*e*_ provides better coverage but increases computational cost. Meanwhile, ${C_d}$ controls the perturbation of observations and the spread of ensemble members. Following previous work^43^, the exact value of $\eta$ is adopted in this study.

Each ensemble ${m_i}$ consists of model parameters $A,z,{x_0},q,~$and $\mu$, which defines the source of the gravity anomaly to be estimated. Prior bounds for each parameter, denoted as ${M_{min}}$ and ${M_{max}}$, constrain the search space for inversion. The initial ensemble members $m_{i}^{{\left( 0 \right)}}$ are randomly generated within the following bounds:

13 $$m_{i}^{{\left( 0 \right)}}={M_{min}}+\left( {{M_{max}} - {M_{min}}} \right)x~~rand,~~i=1,2,..,{N_e}$$

where *rand* generates uniformly random values between 0 and 1. These prior bounds ensured that all solutions remained geologically plausible throughout the inversion process, consistent with the Bayesian framework (section “Ensemble Kalman inversion”). Next, forward modeling is performed to calculate the gravity response (${{{g}}_{{{cal}}}}$) for each ensemble member using Eq. (12). The misfit function quantifies the difference between observed (${{{g}}_{{{obs}}}}$) and calculated gravity (${{{g}}_{{{cal}}}}$) data as a function of the model parameters (*M*). This misfit was evaluated using the RMSE equation, which can be written as follows^5^ :

14 $$R{{MSE~}}\left( {{M}} \right){{=}}\sqrt {\frac{{\mathop \sum \nolimits_{{{{j=1}}}}^{{{N}}} {{\left( {{{{g}}_{{{obs}}}}~{{-~}}{{{g}}_{{{cal}}}}\left( {{M}} \right)} \right)}^{{2}}}}}{{{N}}}}$$

where *N* is the total number of gravity observations (${{{g}}_{{{obs}}}}$).

After the misfit evaluation, the ensemble of model parameters is updated iteratively using the EKI framework. The update step refines the model parameters by incorporating observational information while maintaining ensemble diversity. At each iteration, the statistical parameters in the EKI framework, e.g., the ensemble mean ($\bar{m}$), the mean of the predicted data ($\bar{d}_{{cal}}$), the cross-covariance (${C_{md}}$), and the data covariance (${C_{dd}}$) are computed sequentially using Eqs. (5), (6), (7), and (8). The Kalman gain (*K)* is then calculated using the Tikhonov-regularized formulation to stabilize the inversion process, as expressed in Eq. (10). The choice of the regularization parameter ($\lambda$) in Eq. (10) is critical. If $\lambda$ is too small, regularization may be ineffective. Conversely, if $\lambda$ is too large, the solution may be overly smooth or biased. To systematically assess the influence of $\lambda$ on the inversion results, numerical experiments were performed with trial values of ${10^{ - 5}},{10^{ - 4}},{10^{ - 3}},{10^{ - 2}},{10^{ - 1}},$1, and 10, covering a range from very small to relatively large regularization strengths. The next step is to generate perturbed observations to account for measurement noise (${{g}}_{{{{obs}}}}^{{{j}}}$), following:

15 $$~{{g}}_{{{{obs}}}}^{{{j}}}{{=}}{{{g}}_{{{obs}}}}{{+N}}\left( {{{0,\varvec{\upsigma}}}_{{{\varvec{\upeta}}}}^{{{2}}}} \right)$$

where ${{N}}\left( {{{0,\varvec{\upsigma}}}_{{{\varvec{\upeta}}}}^{{{2}}}} \right)$ represents Gaussian noise with variance ${{\varvec{\upsigma}}}_{{{\varvec{\upeta}}}}^{{{2}}}$. The ensemble members are then updated using the following Kalman update equation:

16 $${{m}}_{{{i}}}^{{{{k+1}}}}{{=m}}_{{{i}}}^{{{k}}}{{+}}{{{K}}^{{k}}}\left( {{{g}}_{{{{obs}}}}^{{{j}}}{{-g}}_{{{{cal,i}}}}^{{{k}}}} \right)$$

After each update, boundary handling is applied to ensure that parameters remain within their prior bounds. Instead of simple truncation, a reflection scheme is used:

17 $$m_{{i,j}}^{{k+1}}{{=}}\left\{ {\begin{array}{*{20}{c}} {2{M_{min,j}} - m_{{i,j}}^{{k+1}}~~~~~~~~~~if~~m_{{i,j}}^{{k+1}}<{M_{min,j}}} \\ {2{M_{max,j}} - m_{{i,j}}^{{k+1}}~~~~~~~~~~~elseif~~m_{{i,j}}^{{k+1}}>{M_{max,j}}} \\ {~~m_{{i,j}}^{{k+1}}~~~~~~~~~~~~~~~~~~~~~~~~~else~~~~~~~~~~~~~~~~~} \end{array}} \right.$$

This reflection strategy prevents parameters from sticking at the boundaries and enhances exploration of the search space while preserving physical plausibility. To further promote convergence, a greedy selection strategy is applied after each iteration. The updated misfit of each ensemble member $\left( {f_{i}^{{new}}} \right)$ is compared to its previous value $\left( {f_{i}^{{old}}} \right)$. If an update produces improvement $\left( {f_{i}^{{new}}<f_{i}^{{old}}} \right)$, the new parameter vector is accepted. Otherwise, the old solution is retained:

18 $$m_{i}^{{k+1}}=\left\{ {\begin{array}{*{20}{c}} {m_{i}^{{k+1}}~~~~~~~~~if~~f_{i}^{{new}}<{{f}}_{{{i}}}^{{{{old}}}}} \\ {m_{i}^{k}~~~~~~~~~~~~~~~~else~~~~~~~~~~~~~~} \end{array}} \right.$$

The combination of reflection-based boundary handling and greedy selection ensures that the inversion progresses consistently toward better solutions while maintaining stability of the ensemble.

In geophysical inversion, estimating model parameter uncertainties ensures a reliable interpretation. The ensemble in the EKI framework estimates these uncertainties using the limit of acceptability and objective function trade-off methods^43,60,61^. As a result, multiple parameter sets can produce acceptable fits, forming the posterior distribution model (PDM)^62^. Using its optimization capabilities, the EKI inversion program is expected to achieve optimal gravity inversion solutions with enhanced performance.

**Figure:**
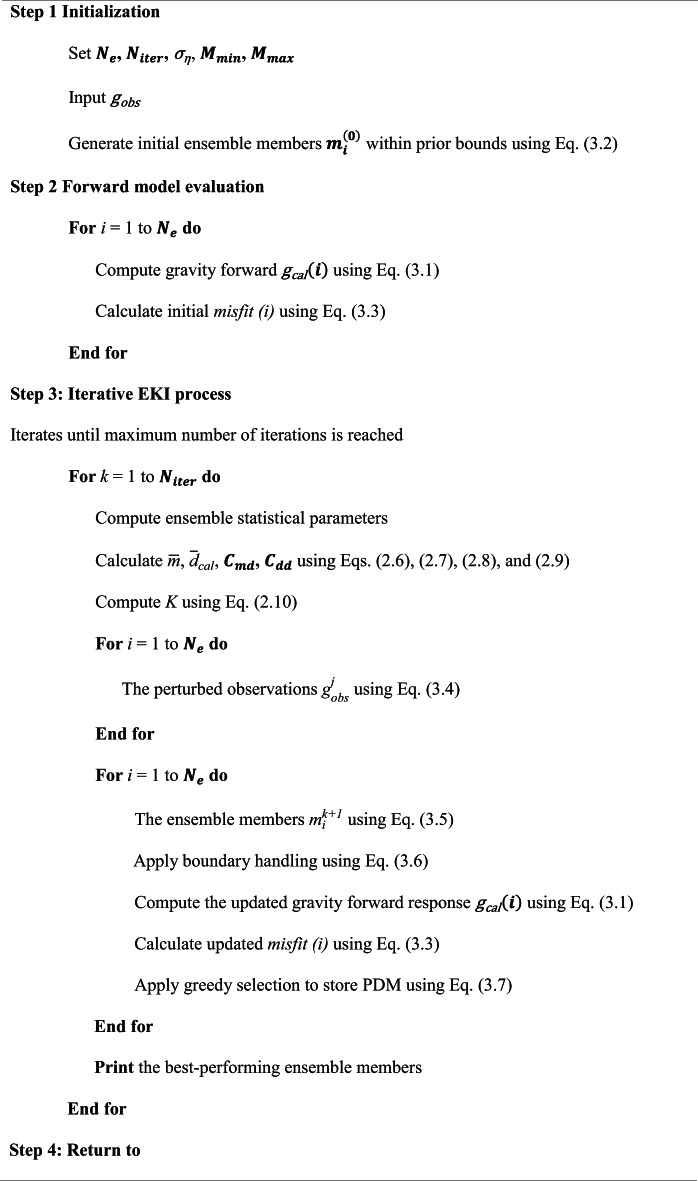
**Algorithm 1**: Pseudocode of the regularized ensemble Kalman inversion (EKI) program for gravity data inversion.

## Synthetic data inversion

To assess the performance, stability, robustness, and efficiency of the proposed regularized ensemble Kalman inversion (EKI), we designed a series of controlled synthetic gravity data inversion experiments. First, a sensitivity analysis was performed to investigate the influence of model parameters on the gravity response. Second, a set of numerical experiments were conducted to systematically examine the effects of ensemble size and the regularization parameter $\left( \lambda \right)$ on the inversion stability and convergence. Third, the EKI’s capacity to recover subsurface parameters was evaluated under both noise-free and noise-contaminated conditions using two scenarios: (1) a single-source anomaly representing a solitary orebody and (2) a multiple-source anomaly simulating the more complex case of overlapping bodies. Finally, the robustness and computational efficiency of the proposed method were benchmarked against well-established gravity inversion algorithms, providing a comparative basis to demonstrate the advantages of EKI.

### Sensitivity analysis

Sensitivity analysis is commonly performed using the Jacobian matrix, typically computed via the finite difference method^63^. In this study, we employ a gradient-free approach^64,65^, which estimates the sensitivity of the gravity response by perturbing individual model parameters and observing the resulting changes in the forward-model outputs. This method circumvents computationally intensive derivative calculations while requiring no explicit gradient information. In this sensitivity test, we independently perturbed each parameter of the two-body gravity model by ± 50% of its true value, while keeping the other parameters fixed. The gravity responses were computed and visualized to qualitatively evaluate the influence of each parameter on the model response (anomaly shape and amplitude). This sensitivity analysis helps interpret inversion uncertainties by identifying the influence of parameters on the gravity response.

**Fig. 2:**
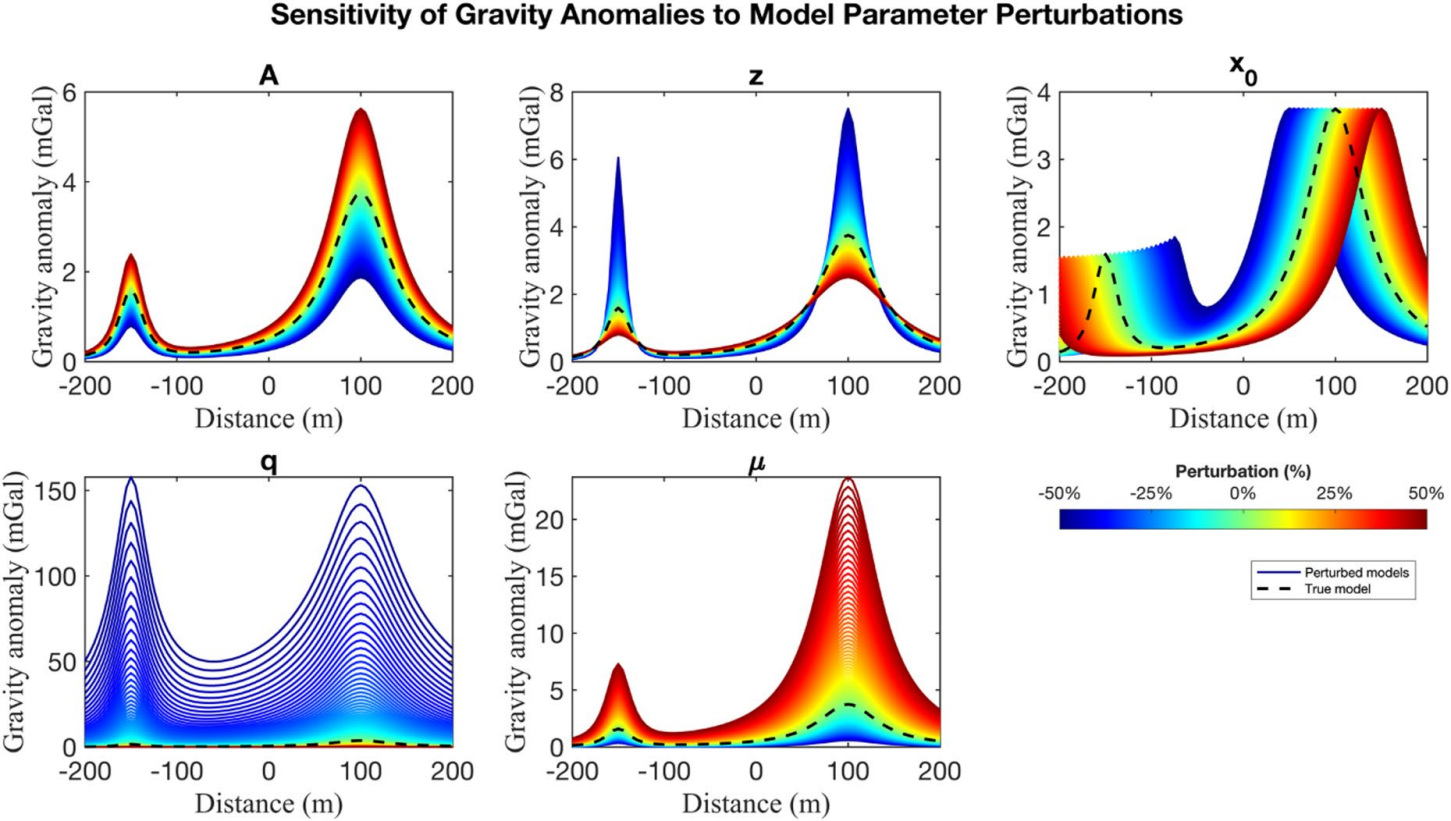
Sensitivity analysis of gravity response to ± 50% perturbation for each model parameter in multiple anomaly cases: A, z, ${x_0}$ (top row); q, µ (bottom row). The black dashed lines represent the gravity response from the unperturbed (true) model. The coloured spectrum indicates how changes in each parameter affect the gravity response.

In Fig. 2, the colour bar illustrates the relative sensitivity of each model parameter to perturbations. The depth parameter *z* showed the strongest effect, as the perturbation significantly altered both the amplitude and shape of the anomaly. The ${x_0}$ parameter also exhibited strong influence, with perturbation directly shifting the anomaly laterally. The shape factor *q* controlled the anomaly curvature, with perturbations resulting in sharper or broader anomaly peaks, reflecting medium-to-high sensitivity. In contrast, the amplitude coefficient *A* acted mainly as a linear scaling factor, modifying the overall magnitude without altering the curve geometry, thus showing lower sensitivity. Similarly, the shape factor $\mu$ influenced the anomaly height but preserved the overall curve shape, making it less sensitive compared to *z*, ${x_0}$, and *q.* These findings demonstrate that gravity data provide strong constraints on location-related and shape parameters but weaker constraints on scaling parameters. The disparity in sensitivity underscores the need for systematic uncertainty analysis to ensure robust outcomes and mitigate interpretational bias.

### Effect of ensemble size and regularization parameter $\left( \varvec{\lambda} \right)$

This section reports numerical experiments investigating the influence of ensemble size and the regularization parameter ($\lambda$) on the performance of the regularized EKI, as both are key determinants of inversion stability and accuracy.

#### Ensemble size

We investigated the effect of ensemble size (*N*_*e*_) on inversion performance and statistical characteristics of the inverted parameters through controlled experiments with a single-anomaly model (Table 2). Seven ensemble sizes (*N*_*e*_ = 50–500) were tested across 30 independent realizations, enabling a robust evaluation of convergence behaviour, the best objective function values, and stochastic sampling variability.

**Table 2:** Statistical characteristics of source parameters, best objective function values, and iterations to convergence for different ensemble sizes across 30 EKI realizations.

Ensemble size (Ne)	Best parameter estimations (median ± interquartile range) across 30 realizations						
	$\:{A}\:\left(\:{m}{G}{a}{l}.{{m}}^{2}\:\right)$	z (m)	$\:{x}_{0}$ (m)	q	$\:{\upmu\:}$	Best Obj (mGal)	Iterations to convergence
50	528.39 ± 106.6	24.96 ± 0.15	-50.00 ± 0.00	0.99 ± 0.00	0.97 ± 0.05	2.7e-03 ± 0.01	407 ± 826
100	544.29 ± 61.3	24.99 ± 0.08	-49.99 ± 0.00	0.99 ± 0.00	0.97 ± 0.03	4.16e-04 ± 0.0	315 ± 453
150	598.65 ± 90.2	24.99 ± 0.00	-50.00 ± 0.00	1.00 ± 0.00	0.94 ± 0.04	4.06e-04 ± 0.0	262 ± 232
200	558.20 ± 116.6	25.00 ± 0.00	-50.00 ± 0.00	1.00 ± 0.00	0.96 ± 0.06	1.95e-04 ± 0.0	235 ±241
300	557.74 ± 111.6	24.99 ± 0.00	-49.99 ± 0.00	1.00 ± 0.00	0.96 ± 0.06	6.17e-05 ± 0.0	176 ± 85
400	557.81± 92.93	24.99 ± 0.00	-50.00 ± 0.00	1.00 ± 0.00	0.95 ± 0.05	7.94e-05 ± 0.0	190 ± 93
500	584.79 ± 98.8	25.00 ± 0.00	-50.00 ± 0.00	1.00 ± 0.00	0.95 ± 0.05	1.22e-04 ± 0.0	128 ± 75
True	500	25	-50	1	1	-	-

**Fig. 3:**
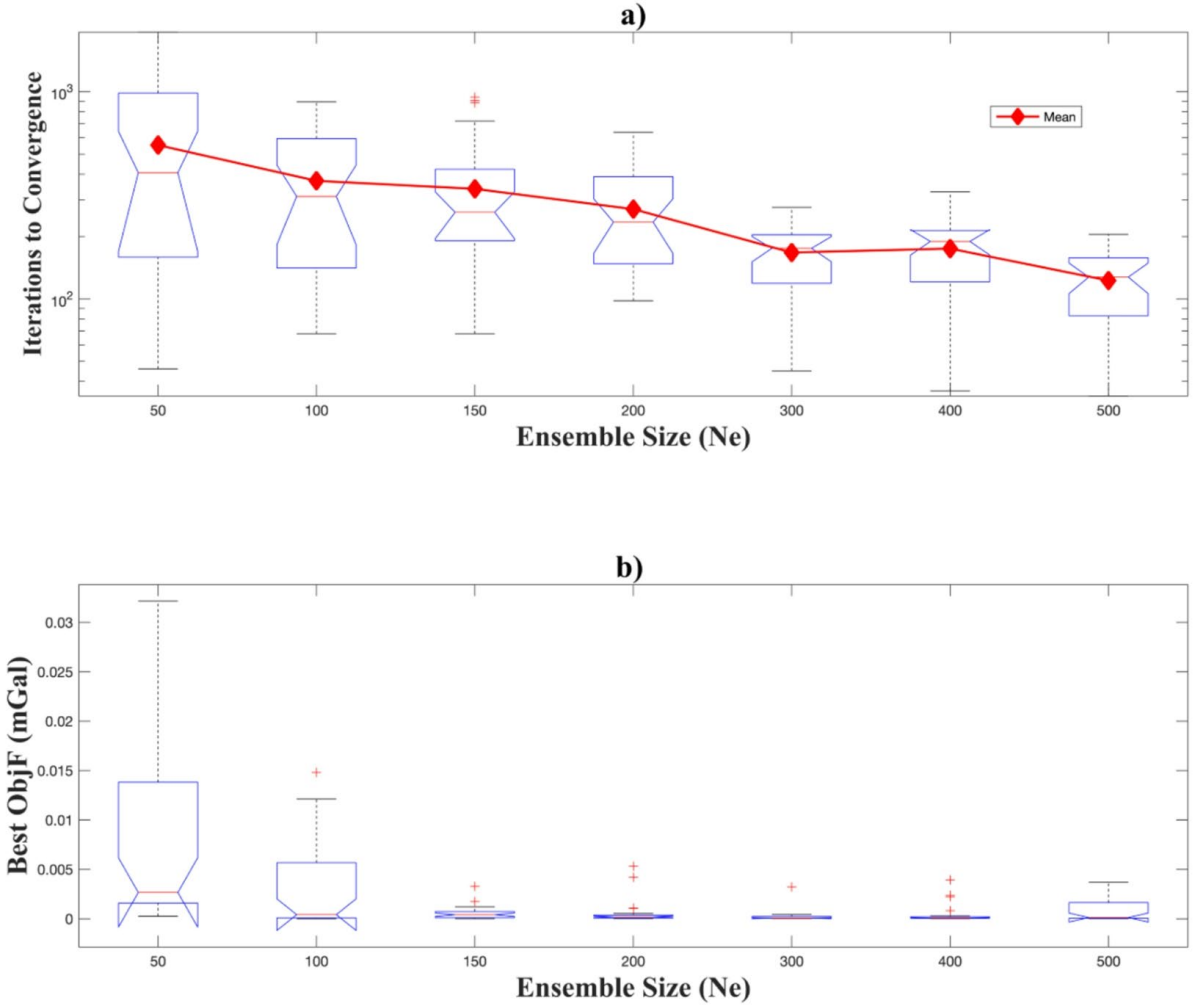
Effect of ensemble size on the EKI results. Box-and-whisker plots summarizing the statistical outcomes from 30 realizations for each ensemble size. (a) The number of iterations to convergence illustrates that larger ensembles significantly accelerate the convergence. (b) Statistical distribution of the best objective function values, showing that larger ensembles yield more stable and consistent low misfit values.

The results reveal a clear dependence of convergence efficiency on ensemble size. For small ensembles ( *N*_*e*_ = 50–100), convergence typically required more than 300–400 iterations, with a wide interquartile range (IQR) indicating strong variability between realizations (Table 2). As *N*_*e*_ increased, the median number of iterations to convergence generally decreased, reaching approximately 128 ± 75 iterations at *N*_*e*_ *=* 500. This trend, with reduced spread across realizations, demonstrates that larger ensembles enhance stability and accelerate convergence, thereby enabling more efficient exploration of the parameter space (Fig. 3a).

The best objective function values across 30 realizations followed a similar trend, decreasing substantially as ensemble size increased. As shown in Table 2, for $\:{N}_{e}\ge\:100$, the best objective function values were relatively close to each other, indicating consistency across realizations. The box-and-whisker plots (Fig. 3b) further illustrate this behaviour: small ensembles ( *N*_*e*_ = 50, 100) produced longer boxes and whiskers, reflecting higher variability in the achieved misfit values. In contrast, larger ensembles (*N*_*e*_ $\:\ge\:$ 150) yielded narrower distributions, with the boxes and whiskers becoming very short and centred near zero, signifying reduced variability and more stable convergence. Nevertheless, the improvement between *N*_*e*_ *=* 100 and *N*_*e*_ = 150 was not strongly pronounced, suggesting that ensemble sizes of around 100–150 are already adequate to achieve optimal results for the single-anomaly case, while larger ensembles may be required for more complex scenarios.

**Fig. 4:**
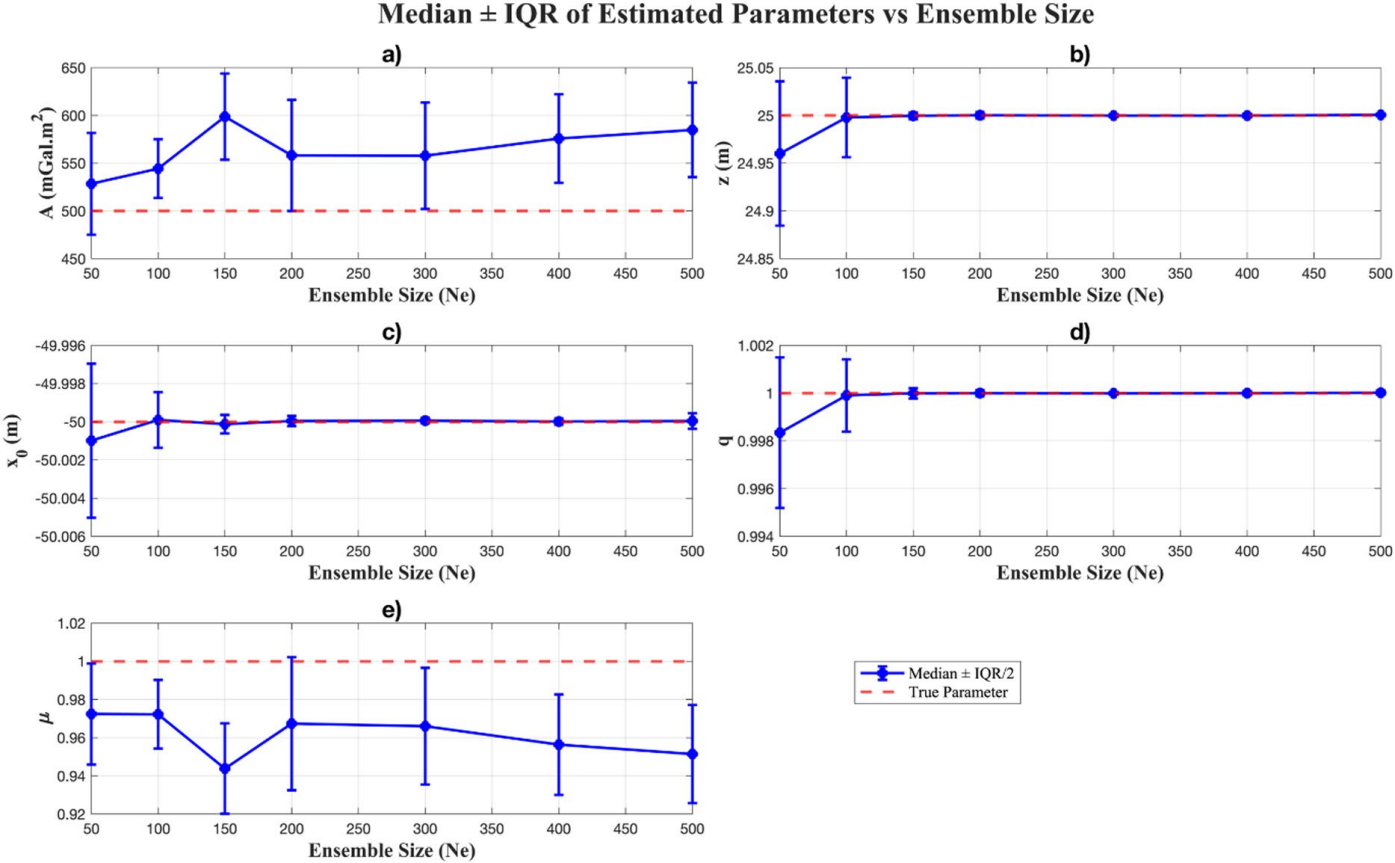
Median and interquartile range (IQR) of the recovered parameters across 30 realizations for different ensemble sizes. Geometric parameters (*z*, ${x_0}$, *q*) are accurately resolved with narrow IQRs (represented by error bar), while scaling-related parameters (*A* and $\mu$) show larger variability and bias, especially at *N*_*e*_ < 100. Larger ensembles reduce uncertainty, though residual bias persists for $\mu$ due to its lower sensitivity.

In terms of parameter recovery (shown in Table 2; Fig. 4), geometric parameters ( e.g., *z*, ${x_0}$, and *q*) were consistently estimated with high accuracy across all ensemble sizes (Fig. 4b – d). Their median values remained very close to the actual values of model parameters, with negligible IQRs, confirming their strong resolvability. In contrast, amplitude *A* and the shape factor $\mu$ (Fig. 4a, e) exhibited systematic deviations from the true values, with broader IQRs particularly at smaller ensembles, indicating higher variability and residual bias. Although increasing ensemble size (*N*_*e*_ $\geqslant$ 100) improved the estimates and reduced posterior spread, but some bias persisted, especially for *A* and $\mu$. This behaviour is consistent with the sensitivity analysis (section “Sensitivity analysis”), which demonstrated that gravity data strongly constrain *z*, ${x_0}$, and *q*, while *A* and $\mu$ remain weakly constrained and therefore more susceptible to ensemble sampling effects and the ill-posedness of the inversion.

From a Bayesian perspective, ensemble size directly governs the quality of posterior approximation in EKI. Small ensembles inadequately sample the parameter space, yielding biased estimates and inflated uncertainty. On the other hand, larger ensembles capture parameter correlations more faithfully, reducing posterior spread and accelerating convergence. Thus, ensemble size represents a trade-off between computational cost and inversion reliability, with larger ensembles offering more robust solutions at a higher expense. These findings provide practical guidance for selecting ensemble sizes in gravity data inversion to balance efficiency and accuracy.

#### Regularization parameter $\varvec{\lambda}$

In our implementation, the regularization parameter *λ* is introduced solely to stabilize the computation of the Kalman gain. Without this term, the auto-covariance matrix in Eq. (9) can become ill-conditioned or singular, preventing inversion. Adding *λI* (Eq. 10) improves the matrix conditioning and ensures that parameter updates remain numerically stable. To systematically assess the effect of the regularization parameter $\lambda$ on the performance of EKI, we conducted numerical experiments covering both single-anomaly and multi-anomaly scenarios, under noise-free and noise-contaminated conditions. Seven $\lambda$ values (1e-5–10) were tested, each with 30 independent realizations to account for stochastic variability in the ensemble initialization and to ensure robust statistical evaluation. Each realization used an ensemble size of *N*_*e*_ = 150 and a maximum of 200 iterations. Two primary metrics were analysed: the success rate (percentage of runs converging within the iteration limit) and the best objective function value (minimum data misfit achieved among all realizations) for a given $\lambda$. Median and interquartile range (IQR) of the objective function were further calculated to provide a statistically robust characterization of stability and convergence behaviour.

**Fig. 5:**
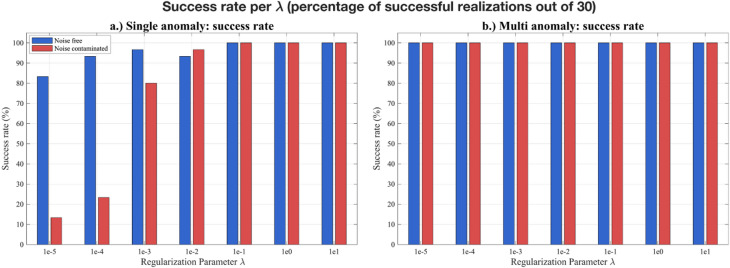
Success rate of different regularization parameters ($\lambda$) over 30 realizations in each scenario. (a) single anomaly; (b) multi-anomaly. Blue and red bars correspond to noise-free and noise-contaminated scenarios, respectively.

The success rate serves as an initial indicator of the algorithm’s stability across varying lambda values. In the single-anomaly noise-free case, EKI achieved a high success rate (> 90%) for $\lambda {{~}} \geqslant 1{{e}} - 4$, and reached 100% success when $\lambda {{~}} \geqslant 0.1$ (Table 3). At $\lambda$ = 1e-5, the stability is slightly reduced (83% success rate), with several realizations exhibiting complex objective function values, indicating ill-conditioned covariance matrices and oscillatory ensemble updates. Under noise-contaminated conditions, convergence became much more sensitive to $\lambda$. The success rate was only 13–23% for $\lambda {{~}} \leqslant 1{{e}} - 4$ but sharply increased to 80% at $\lambda {{~}}={{~}}1{{e}} - 3$, and reached 100% success rate for $\lambda {{~}} \geqslant 0.1$. When comparing the two conditions (Fig. 5), the noise-free case consistently achieved higher success rates than the noise-contaminated case. This trend demonstrates that adequate regularization suppresses noise amplification and maintains stable ensemble updates in the presence of measurement uncertainty. In contrast, all multi-anomaly scenarios maintained 100% success across all $\lambda$ values (both noise-free and noise-contaminated conditions), suggesting that the stronger spatial structure of the model imposed an additional constraint that enhanced stability. This indicates that increasing model complexity and the presence of noise did not reduce stability, suggesting that EKI is relatively insensitive to $\lambda$ variations in such settings. To further examine this behaviour, the distribution of best objective function values was analysed next.

**Fig. 6:**
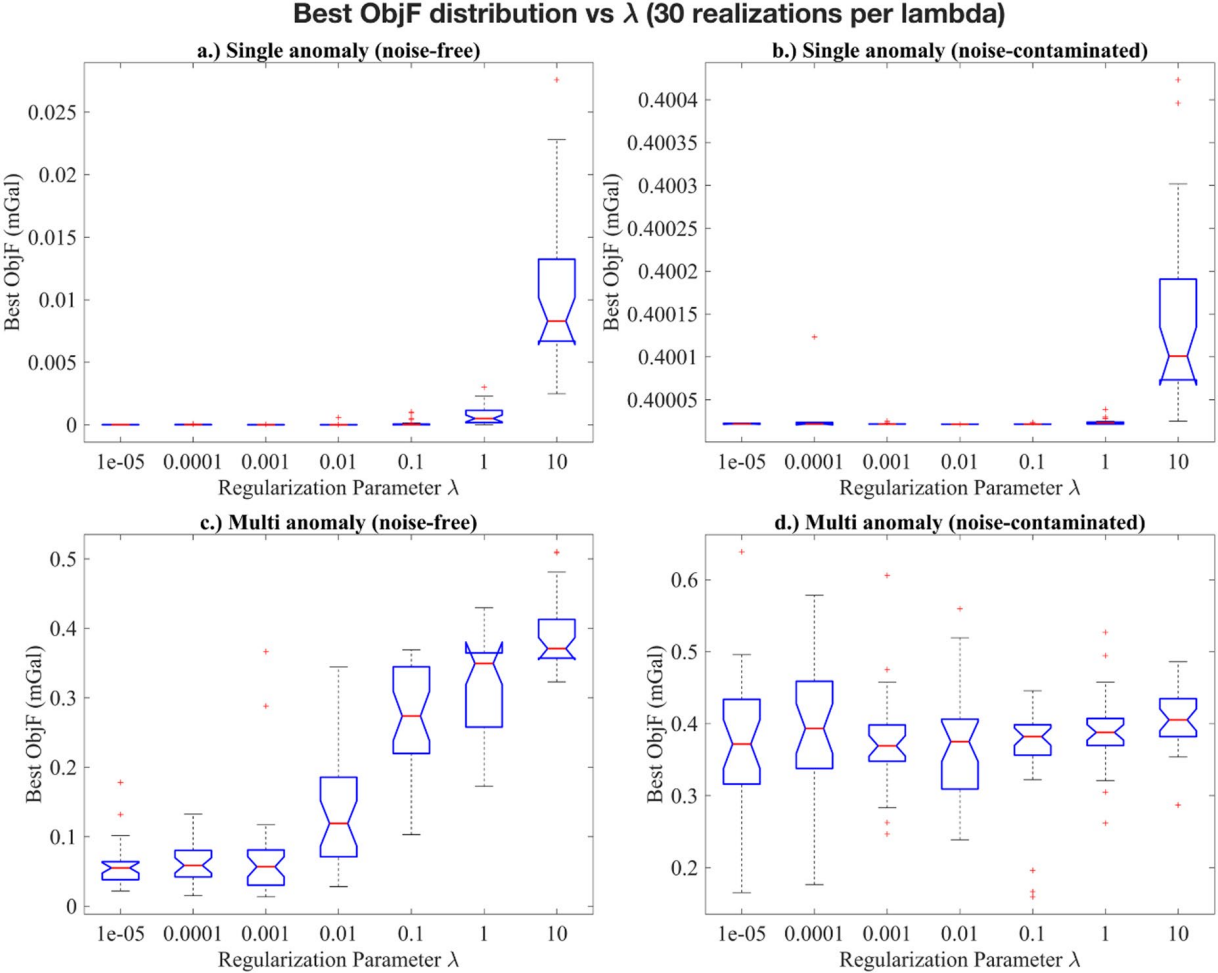
Distribution of the best objective function values for different regularization parameters ($\lambda )$ over 30 realizations per scenario. a) Single anomaly (noise-free); b) Single anomaly (noise-contaminated); c) Multi anomaly (noise-free); d) Multi anomaly (noise-contaminated).

The distribution of the best objective function values provides further insight (Table 3; Fig. 6). For the single-anomaly noise-free case, the median of the objective functions remained low and consistent for $1e - 5~ \leqslant \lambda \leqslant 1e - 2$, with increased variability at $\lambda \geqslant 0.1$. This pattern suggests that moderate regularization promotes convergence toward the global minimum, while excessive regularization leads to over-smoothing and reduces data-fitting capability. Under noise contamination, the median of the objective function is consistent at around 0.40 for all $\lambda \geqslant 1e - 3$, with the narrow IQRs (Fig. 6), indicating that appropriate regularization effectively suppresses noise amplification and yields consistent results across different realizations. At $\lambda \leqslant 1e - 4$, the objective function values distribution became erratic and exhibited occasional outliers, consistent with the reduced success rates and numerical instability discussed earlier. For the multi-anomaly case, the median objective function gradually increased with $\lambda$, reflecting the influence of regularization strength on inversion smoothness. All realizations converge with compact IQRs, confirming stable and well-conditioned performance. Similar behaviour was observed under noise contamination, with slightly higher median objective function yet comparable variability.

Overall, these results demonstrate that the moderate regularization $\left( {1e - 3 \leqslant \lambda \leqslant 1e - 1} \right)$, provides the best balance between stability and resolution. Excessively small $\lambda$ value leads to under-regularization and unstable behaviour, whereas overly strong regularization ($\lambda \geqslant 1)$ tends to over-dampen parameter updates, increasing residuals through over-smoothing. As a consequence, $\lambda$ primarily functions as a numerical safeguard in the Kalman gain computation rather than a tuning parameter for data fitting. Selecting $\lambda ~$within a moderate range ensures robust, well-conditioned, and geologically plausible inversion results.

**Table 3:** Performance summary of 30 independent realizations for different regularization parameter $\left( {{\varvec{\uplambda}}} \right)$ across all scenarios, showing success rates and the median of the objective function.

Scenario	${{\varvec{\uplambda}}}$	Realization success	Success rate (%)	Median ObjF (mgal)
Single anomaly noise-free	1e-5	25	83.3	1.38 × 10^− 5^
	1e-4	28	93.3	1.71 × 10^− 5^
	1e-3	29	96.7	9.75 × 10^− 6^
	1e-2	28	93.3	7.35 × 10^− 6^
	1e-1	30	100	2.23 × 10^− 5^
	1	30	100	5.09 × 10^− 4^
	10	30	100	8.30 × 10^− 3^
Single anomaly noise-contaminated	1e-5	4	13.3	0.40
	1e-4	7	23.3	0.40
	1e-3	24	80	0.40
	1e-2	29	96.7	0.40
	1e-1	30	100	0.40
	1	30	100	0.40
	10	30	100	0.40
Multi anomaly noise-free	1e-5	30	100	0.0551
	1e-4	30	100	0.0587
	1e-3	30	100	0.0568
	1e-2	30	100	0.1191
	1e-1	30	100	0.2738
	1	30	100	0.3500
	10	30	100	0.3710
Multi anomaly noise-contaminated	1e-5	30	100	0.3717
	1e-4	30	100	0.3933
	1e-3	30	100	0.3692
	1e-2	30	100	0.3751
	1e-1	30	100	0.3820
	1	30	100	0.3880
	10	30	100	0.4053

### Single-anomaly scenario

The first scenario involves a vertical cylinder with predefined parameters (Table 4). Synthetic gravity data were generated along a linear profile and inverted using the regularized EKI framework (section “Methodology”). Two cases were tested to assess robustness: one with noise-free data and another where the synthetic data were perturbed with 10% Gaussian noise. The prior bounds described in section “Gravity data inversion using EKI” define the search space for inversion. Parameter estimates are reported as the median±interquartile range (IQR) across the ensemble members, providing robust measures of central tendency and dispersion while accounting for inversion uncertainty. In this case, an ensemble size of *N*_*e*_ = 100 and ${N_{iter}}$ = 200 iterations were used to balance convergence and computational efficiency.

**Table 4:** The true values, search space, and inversion results for a single source anomaly using EKI with noise-free and 10% Gaussian noise-contaminated data.

Model parameters	True	Search space	Noise free	Noise contaminated
$A~\left( {{\text{~mGal}}.{{\text{m}}^2}{\text{~}}} \right)$	500	200–700	501.41 ± 12.36	542.79 ± 75.41
*z* (m)	25	10–50	24.99 ± 0.03	22.45 ± 0.75
${x_0}$ (m)	-50	-200–200	-50.00 ± 0.00	-49.86 ± 0.06
*q*	1	0.5–1.5	0.99 ± 0.00	0.92 ± 0.01
$\mu$	1	0–1	0.99 ± 0.01	0.80 ± 0.07
Minimum objective function (mGal)			9.67e-06	0.40
The median objective function (mGal)			2.42e-04	0.40
The interquartile range of the objective function (mGal)			4.74e-04	3.16e-04

Table 4 summarizes the true model parameters, search spaces, and inversion results. In the noise-free case, the recovered parameters were nearly identical to the true values with tight uncertainty bounds, confirming the accuracy of the EKI method. In the noise-contaminated case, the estimated parameters remain close to the true values, though with wider uncertainty bounds, particularly for the amplitude *A* and the shape factor *µ*, which are more sensitive to noise.

**Fig. 7:**
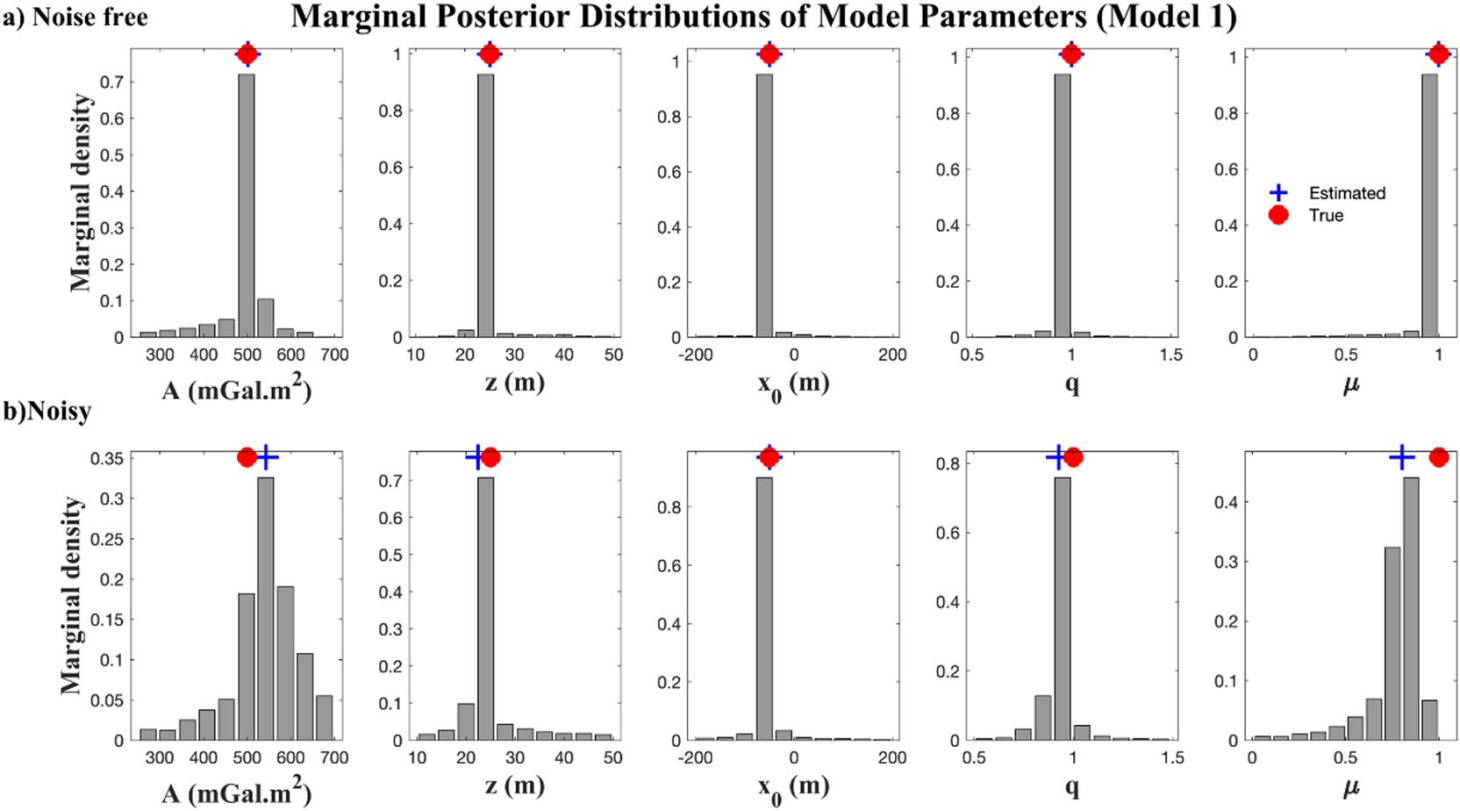
Marginal posterior distributions of parameters for a single anomaly case estimated using EKI: (a) noise-free data; (b) 10% noise-contaminated data.

Figure 7 illustrates the marginal posterior distributions of the model parameters. In the noise-free case (Fig. 7a), the narrow and symmetric distributions indicate a strong constraint. In the noisy case (Fig. 7b), the distributions broadened, especially for *µ*, reflecting increased uncertainty. These histograms highlight parameter identifiability: depth *z* and location $\:{x}_{0}$​ remain well-constrained, whereas shape factors are more sensitive to data quality.

**Fig. 8:**
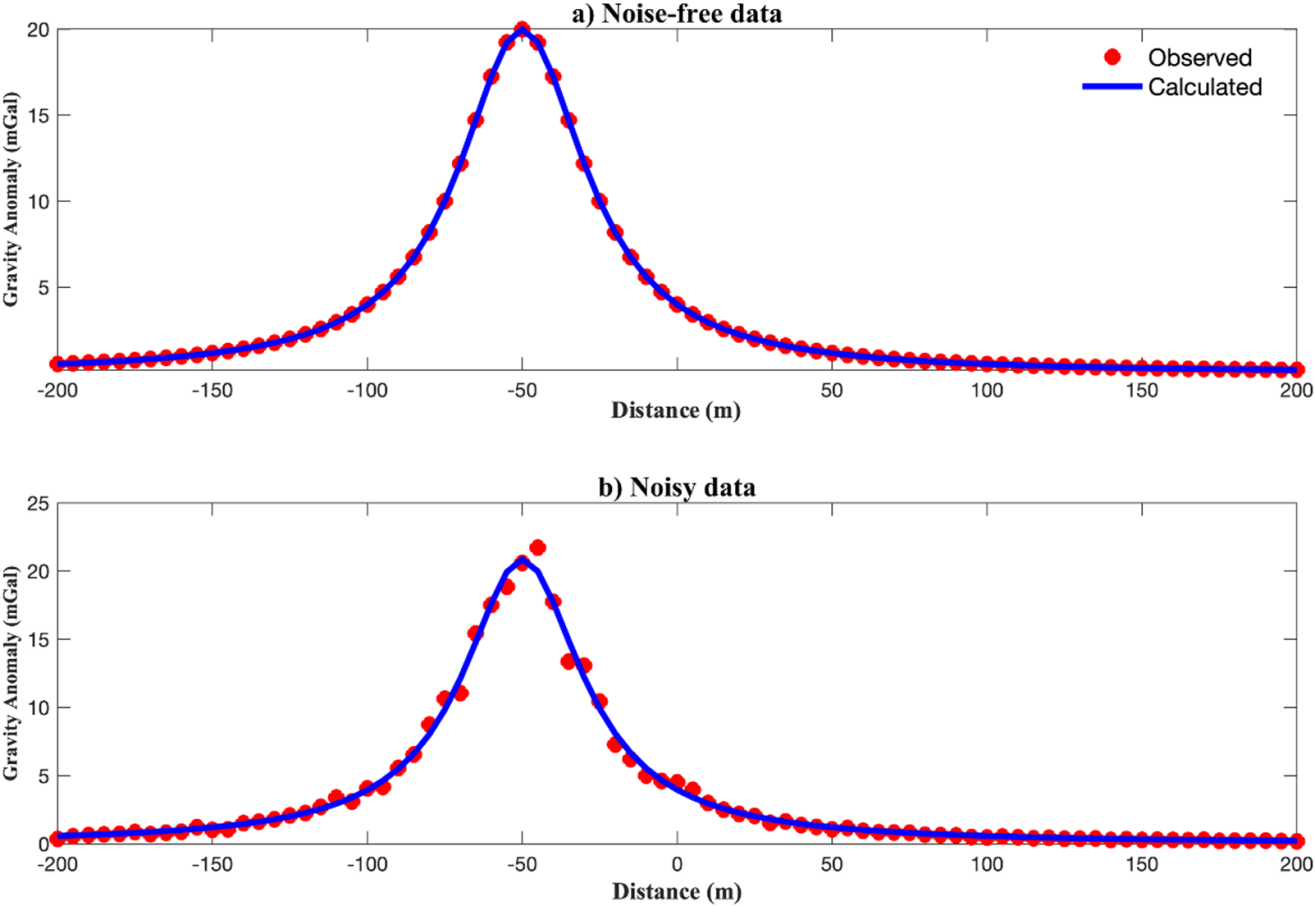
Best-fit gravity curve compared to the observed synthetic data for a single anomaly: (a) noise-free data; (b) noisy data.

**Fig. 9:**
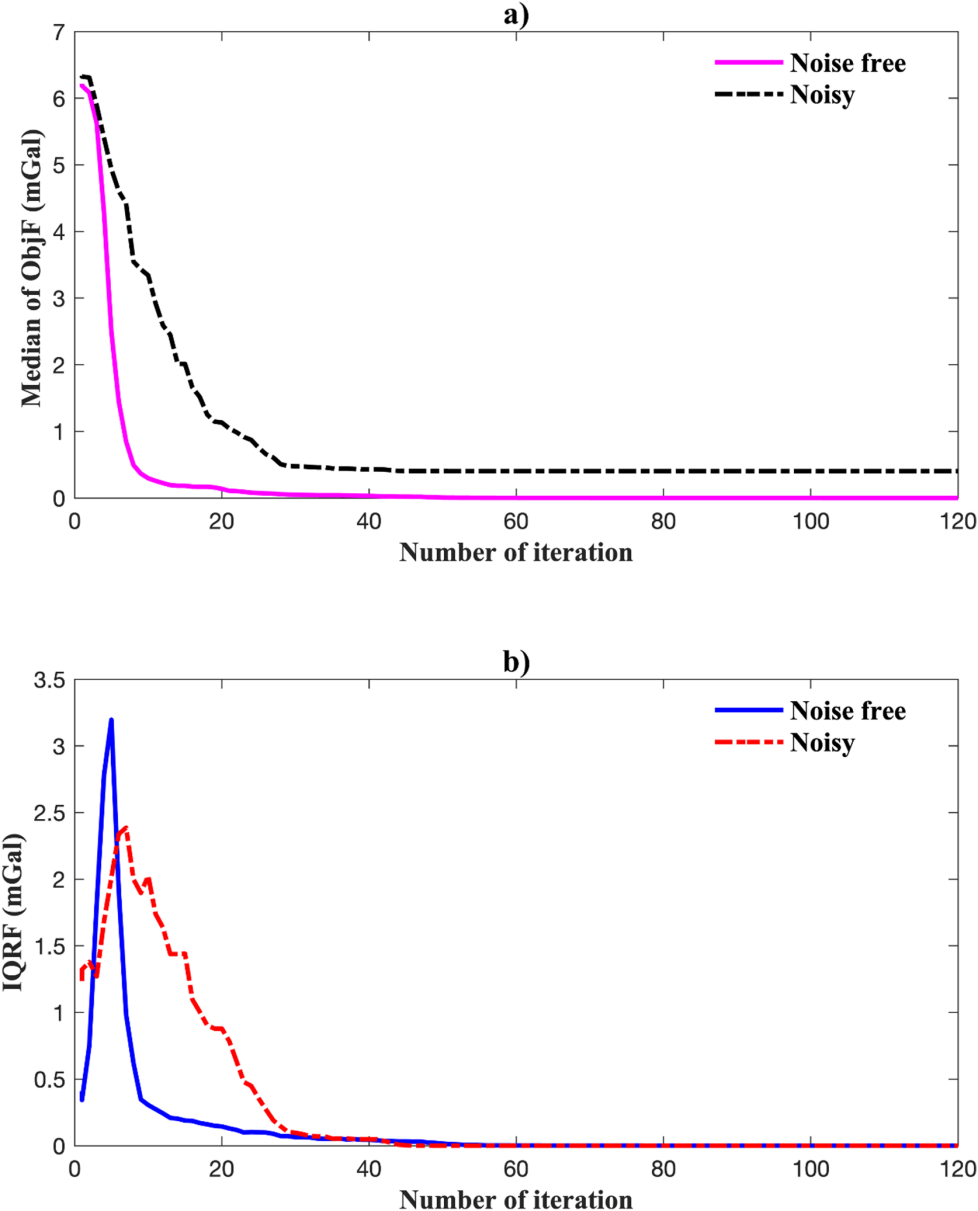
Convergence performance of EKI for a single anomaly: (a) evolution of median RMSE; (b) evolution of the interquartile range (IQR).

Figure 8 shows the gravity anomaly fit using the best-fit model. EKI accurately reproduces the observed data in both the noise-free (Fig. 8a) and noisy (Fig. 8b) cases. Figure 9 shows the inversion’s convergence behaviour. In the noise-free case, the median objective function rapidly decreased and stabilized within 100 iterations, accompanied by a narrowing IQR, indicating consistent ensemble contraction. In the noisy case, convergence was slower and less sharp, but the median and IQR remained stable, demonstrating robustness under data uncertainty.

### Multiple anomaly scenario

A second experiment was performed to simulate more complex geological settings using two overlapping anomalies: a sphere and a horizontal cylinder (Table 5). This case involved estimating 10 parameters, increasing the nonlinearity and complexity of the inverse problem. The noise-free and 10% noise-contaminated data were considered. Given the increased dimensionality, a larger ensemble size (*N*_*e*_ = 200) and longer iterations (${N_{iter}}$ = 4000) were used to ensure adequate exploration of the parameter space and convergence.

**Table 5:** True values, search space, and inversion results for dual anomaly case using EKI with noise-free and 10% Gaussian noise-contaminated data.

Model parameters	True	Search space	Noise free	Noise contaminated
${A_1}\left( {{\text{~mGal}}.{{\text{m}}^2}{\text{~}}} \right)$	600	400–700	639.22 ± 18.70	640.45 ± 36.21
${z_1}\left( m \right)$	20	10–40	19.73 ± 0.38	20.98 ± 0.44
${x_{01}}\left( m \right)$	-150	-200–200	-149.99 ± 0.01	-149.77 ± 0.05
${q_1}$	1.5	0.5–1.5	1.47 ± 0.03	1.49 ± 0.13
${\mu _1}$	1	0–1	0.91 ± 0.08	0.99 ± 0.29
${A_2}\left( {{\text{~mGal}}.{{\text{m}}^2}{\text{~}}} \right)$	150	50–300	212.66 ± 19.89	247.07 ± 20.56
${z_2}\left( {\text{m}} \right)$	40	20–80	40.04 ± 0.04	42.32 ± 0.27
${x_{02}}\left( m \right)$	100	-200–200	99.99 ± 0.00	100.36 ± 0.03
${q_2}$	1	0.5–1.5	1.00 ± 0.00	1.04 ± 0.00
${\mu _2}$	1	0–1	0.90 ± 0.02	0.96 ± 0.03
Minimum objective function (mGal)			7.68e-05	0.14
The median objective function (mGal)			2.48e-04	0.14
The interquartile range of the objective function (mGal)			1.10e-04	1.29e-05

The inversion results are presented in Table 5. In the noise-free case, the estimated parameters closely match the true values with low uncertainty across most parameters. Under noise contamination, the inversion still performed reliably, though uncertainty increases, particularly for parameters related to the second anomaly, such as ${x_{02}},~{q_2}$, ${\mu _2}$​. This reflects the challenges of resolving overlapping anomalies with noisy data, especially when their responses interfere nonlinearly.

**Fig. 10:**
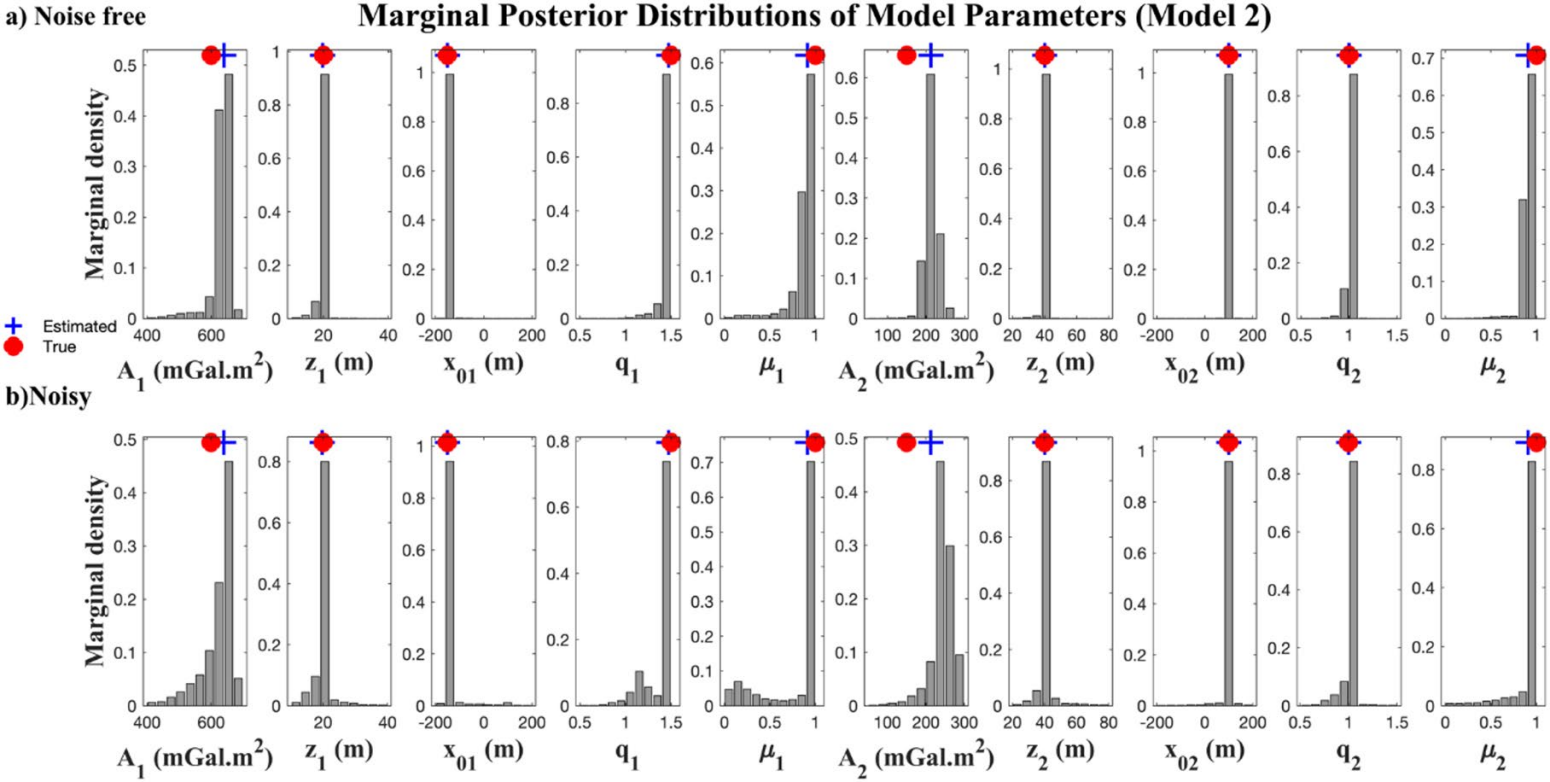
Marginal posterior distribution of parameter distributions for multiple anomaly scenarios using EKI: (a) noise-free data; (b) 10% noise-contaminated data.

The posterior distributions presented in Fig. 10 provide further insights into the inversion process. In the noisy case, the distributions broaden and exhibit some skewness, especially for the shape parameters. However, the medians remain close to the ground truth. These results confirm that the EKI framework, enhanced by Tikhonov regularization and boundary constraints, can provide meaningful uncertainty quantification even in high-dimensional inverse problems.

**Fig. 11:**
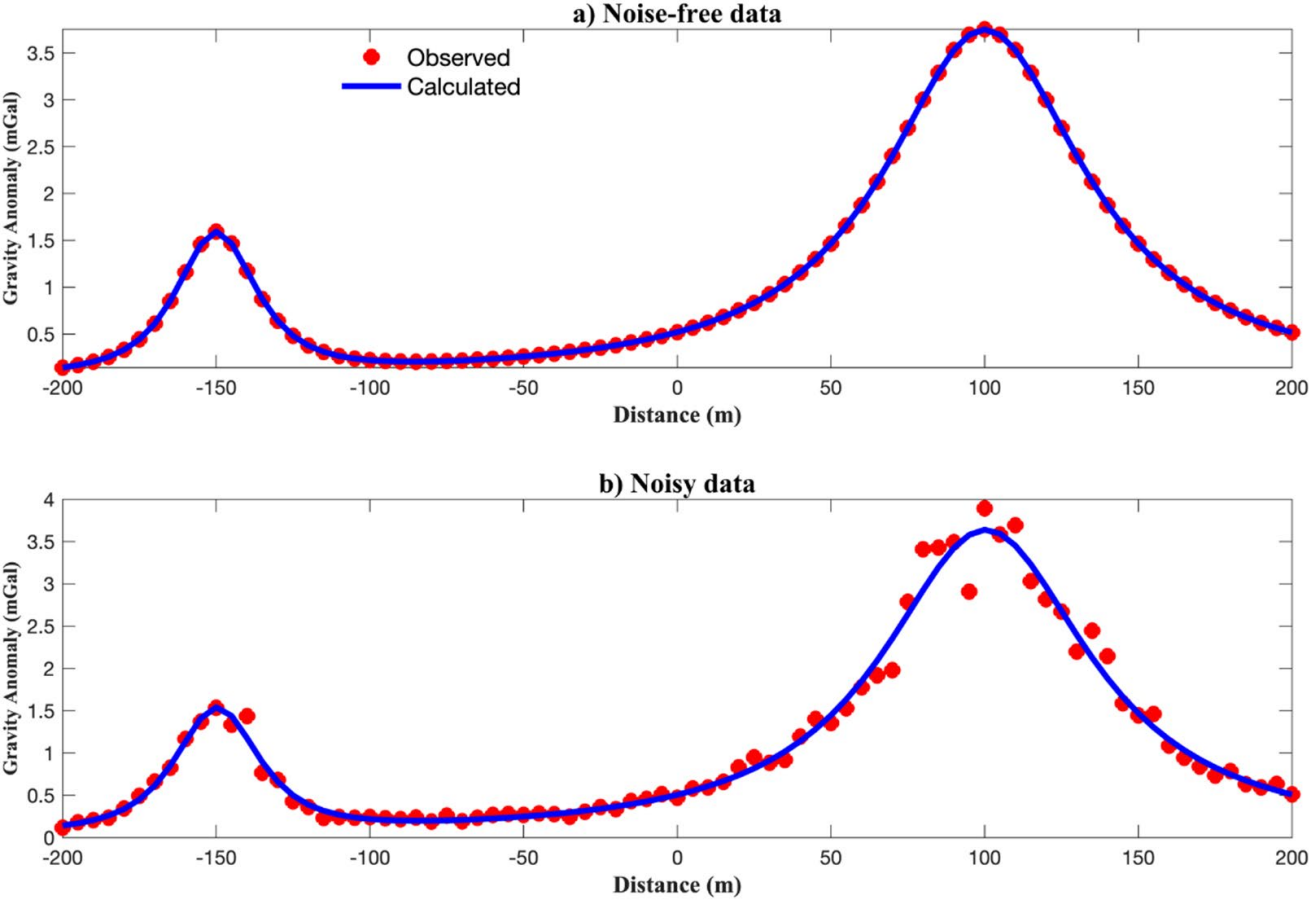
Best-fit gravity curves compared to observed synthetic data for multiple anomalies: (a) noise-free data; (b) noisy data.

**Fig. 12:**
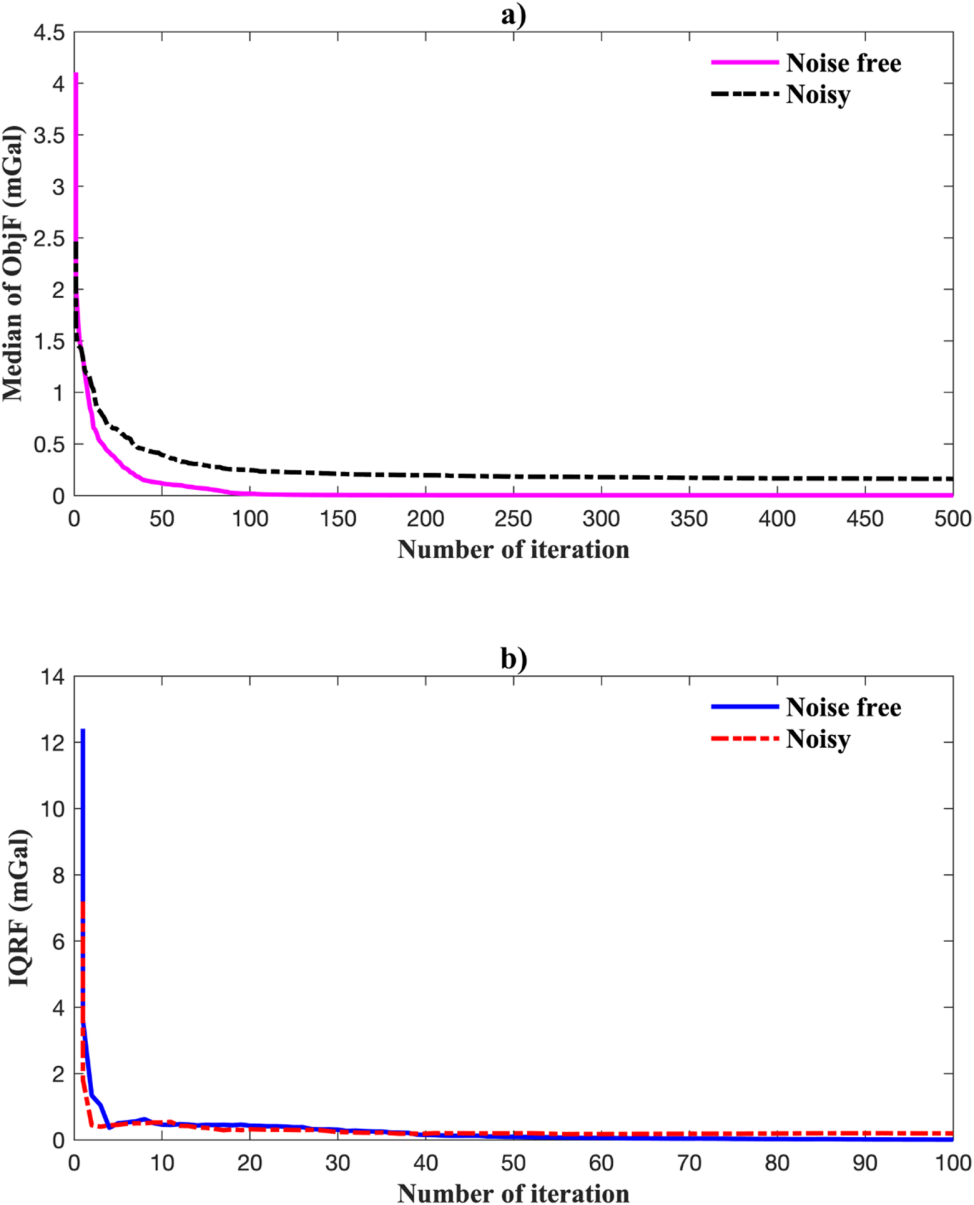
Iterative convergence of EKI for multiple anomalies: (a) median RMSE; (b) IQR of the objective function.

Figure 11 compares the observed and calculated gravity data using the best-fit model. EKI successfully recovered the composite anomaly in both noisy conditions, reproducing its shape and amplitude with high fidelity. The convergence performance shown in Fig. 12 further supports this. The median and IQR of the objective function steadily decreased, with the IQR showing a clear contraction, indicating ensemble stabilization. The choice of a larger ensemble and more iterations was validated by this behaviour, which enabled the EKI algorithm to resolve complex source geometries despite the non-uniqueness and noise.

The results of this comprehensive set of synthetic tests demonstrate that the regularized EKI method is a stable and efficient tool for gravity inversion. The capacity to handle uncertainty and resolve overlapping anomalies makes it a promising approach for real-world geophysical exploration problems.

### Robustness and efficiency of EKI: benchmarking against established algorithms

To quantitatively assess the robustness and computational efficiency of the proposed ensemble Kalman inversion (EKI), we performed a benchmarking experiment against three established metaheuristic algorithms for gravity data inversion: particle swarm optimization (PSO), very fast simulated annealing (VFSA), and the bat algorithm (BA). Each algorithm was implemented using the parameter settings and procedural schemes reported in previous studies, such as– PSO^66,67^, VFSA^14,16^, and BA^3^, to ensure fairness and reproducibility. The inversion was considered successful when the objective function (Eq. 14) dropped below 0.01 mGal, with a maximum of 10,000 iterations (except VFSA, which used 100 × 10,000 iterations). Both PSO and BA employed a population size of 100, while EKI used an ensemble size *N*_*e*_ *=* 100. This benchmark was not intended to identify a universally superior algorithm, but rather to assess the relative robustness and efficiency of EKI compared to established inversion methods.

**Fig. 13:**
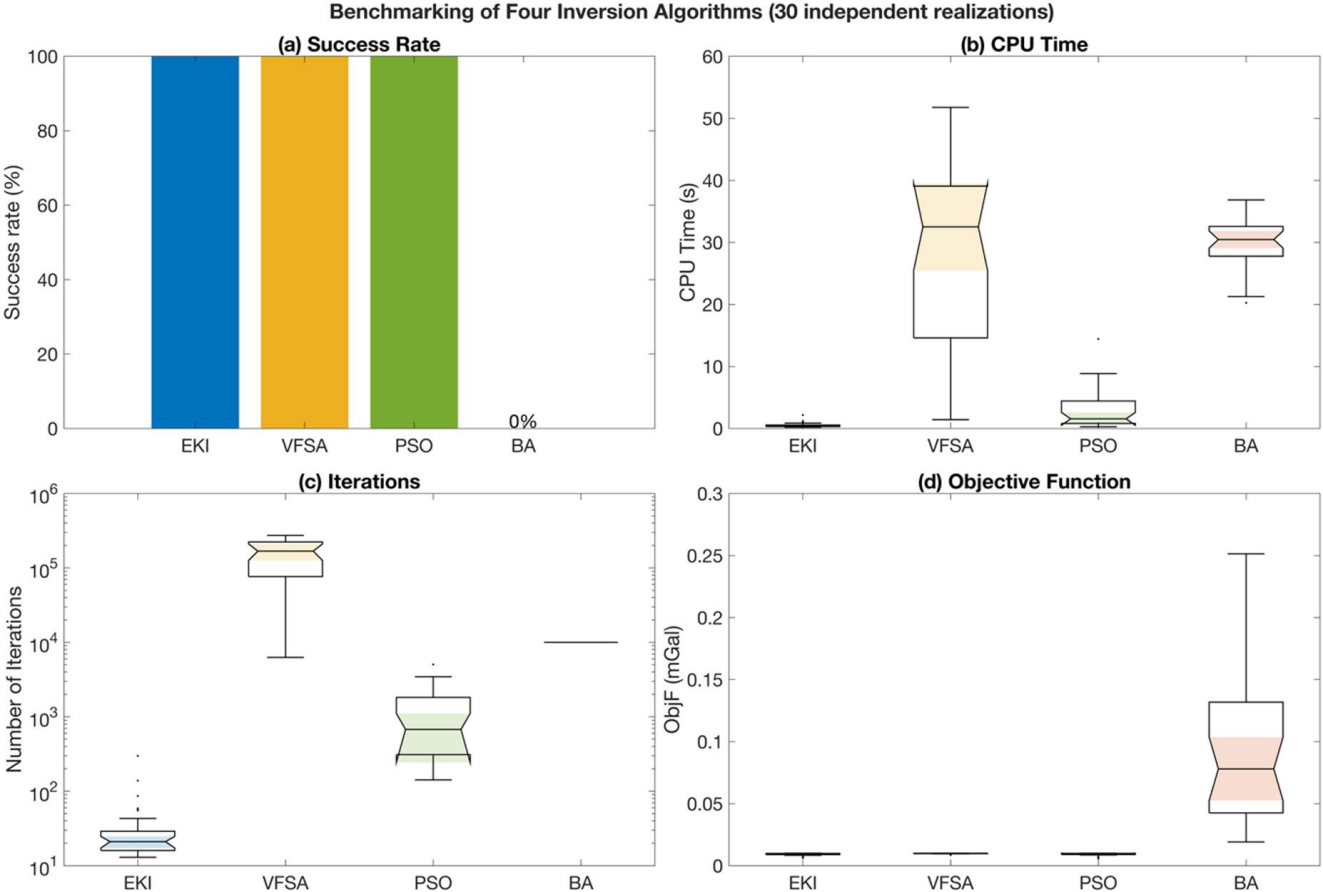
Benchmarking of the proposed ensemble Kalman Inversion (EKI) against established stochastic optimization algorithms. Performance comparison of four algorithms (EKI, VFSA, PSO, and BA) based on 30 independent realizations: (a) success rate, (b) CPU time, (c) number of iterations (log scale), (d) Best objective function values.

The benchmarking results are summarized in Table 6; Fig. 13. The proposed EKI consistently demonstrates superior robustness and computational efficiency across 30 independent realizations. It achieved a 100% success rate with a median CPU time of only 0.38 s and a median of 21 iterations to convergence, demonstrating rapid and stable convergence behaviour. In contrast, VFSA also achieved full convergence success but required significantly higher computational effort, with median CPU time exceeding 32 s and more than $1.6 \times {10^5}$ iterations. PSO showed similarly reliable convergence (100% success rate) but was moderately slower than EKI, whereas BA failed to converge in all realizations under the same stopping criteria. Interestingly, although BA performed effectively in a previous study^3^, it failed to reach the convergence criteria in this benchmark. This discrepancy likely arises from differences in the objective function, consistent with *the No Free Lunch* (NFL) *theorem*, which states that no optimization algorithm can outperform others across all problem landscapes^68^. Despite these variations, all successful algorithms (EKI, VFSA, PSO) achieved comparable final objective function values (~ 0.0095–0.0098), suggesting that the main differences among them lie in robustness and computational efficiency rather than solution quality.

In summary, the benchmarking experiment demonstrates that EKI achieves a remarkable balance between computational efficiency and convergence stability compared to established metaheuristic algorithms. These results highlight EKI’s potential as a robust and efficient inversion algorithm for gravity modelling. Nevertheless, since the benchmark was performed under controlled synthetic conditions, further validation using real field data is required to evaluate EKI’s practical applicability and performance under realistic geological and noise conditions. The following section presents the application of the proposed EKI to field gravity data.

**Table 6:** Statistical summary of benchmarking results for four inversion algorithms (EKI, VFSA, PSO, and BA) over 30 independent realizations.

Inversion algorithm	Success rate (%)	Median CPU time (s)	Median number of iterations	Median ObjF (mGal)
EKI	100	0.3803	21	0.009559
VFSA	100	32.5085	168,205	0.009886
PSO	100	1.5551	677	0.009778
BA	0	30.4623	10,000	0.078016

## Inversion of field data examples

To further validate the performance and applicability of the proposed regularized ensemble Kalman inversion (EKI) under real-world conditions, EKI was applied to four field gravity anomaly datasets associated with known mineralized regions: (1) the Camaguey chromite orebody in Cuba, (2) the Faro Pb-Zn deposit in Canada, (3) the Mobrun sulfide orebody in Canada, and (4) the Mundiyawas Cu-Au deposit in India. Each site represents a distinct geological setting and level of complexity, providing a diverse set of test cases to assess EKI’s robustness, accuracy, and capabilities for quantifying uncertainty. For all field datasets, the inversion results are presented alongside previous studies using metaheuristics and local inversion methods.

### Camaguey chromite orebody, Cuba

**Fig. 14:**
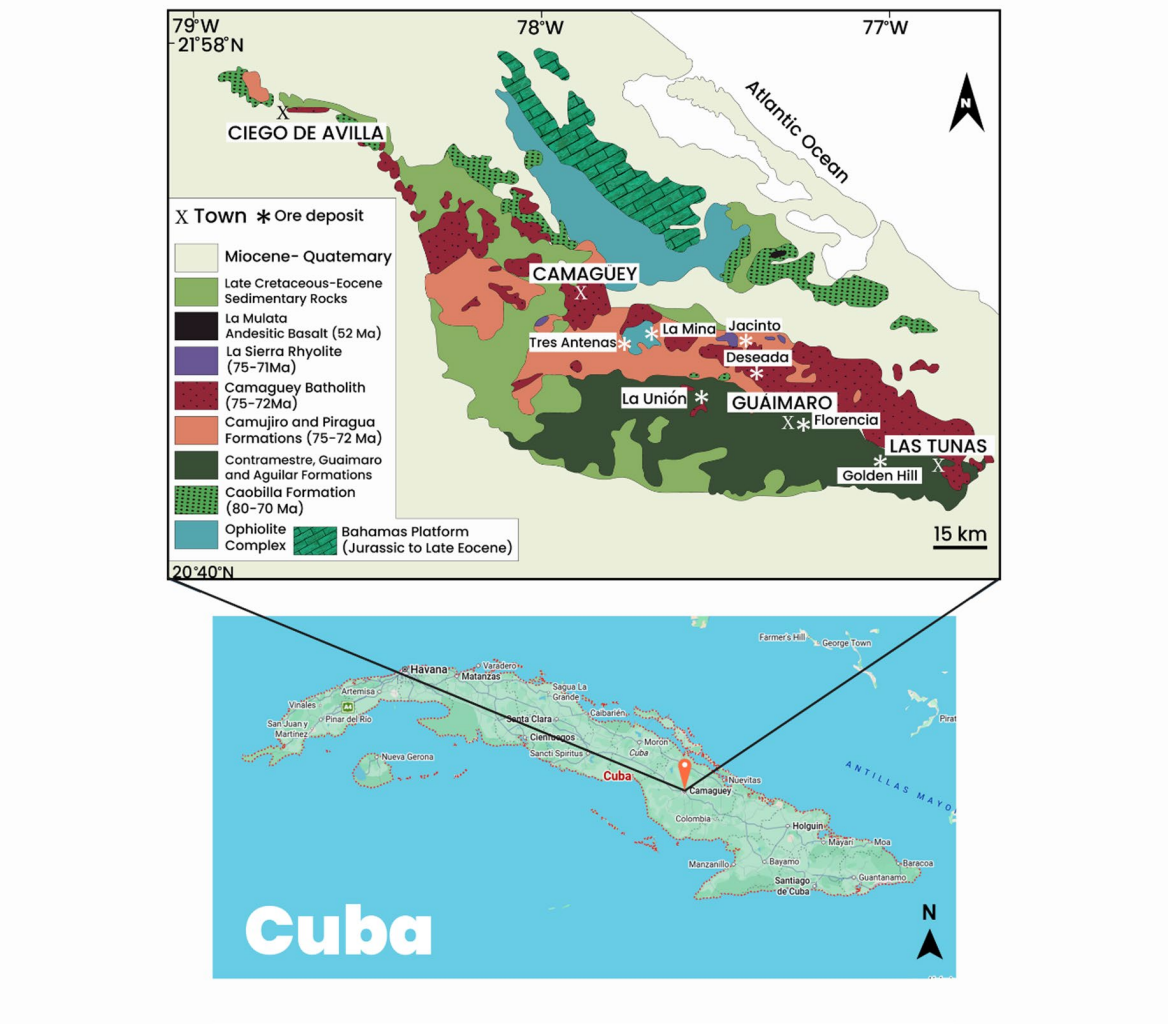
Location and geological map of the Camaguey chromite deposit in Cuba (redrawn after Santana et al.,^70^ using Adobe Illustrator CC 2024 (https://www.its.ac.id/dptsi/en/licensed-software/) under institutional license from Institut Teknologi Sepuluh Nopember).

The Camaguey chromite deposit in Cuba was hosted within a serpentinized ultramafic complex comprising peridotite and dunite units known to concentrate chromite mineralization (Fig. 14). The USGS gravity surveys revealed a prominent anomaly over a ~ 89-meter-long profile (A2B2), which was interpreted to represent a dense chromite-bearing body, as shown in^69^.

**Table 7:** Search space and inversion results for the Camaguey gravity anomaly using the regularized EKI method compared to other methods in previously published studies.

Model parameter	$A~\left( {{\text{~mGal}}.{{\text{m}}^2}{\text{~}}} \right)$	z (m)	z (m)	q	$\mu$	RMSE (mGal)
Search space	1–5	10–20	-50–38	0.5–1.5	0–1	
**EKI**	**4.06 ± 0.61**	**15.10 ± 1.1**	**-1.4 ± 0.05**	**1 ± 0.08**	**0.90 ± 0.22**	**0.0105**
DL Rao (Warnana et al.^5^)	4.97 ± 0.2	16 ± 0.14	-1.5 ± 0.01	1.08 ± 0.02	1 ± 0.03	0.0100
BA (Essa and Diab^3^)	3.3 ± 0.28	16.4 ± 0.45	-1 ± 0.42	1 ± 0.0	1 ± 0.03	0.0121
DE (Ekinci et al.^7^)	3.51	16.52	58.82	1	1	0.0680
VFSA (Biswas^16^)	3.5 ± 0.0	16.2 ± 0.0	-1.8 ± 0.0	1	-	0.0081
R imaging (Essa et al.^8^)	1.86	16	0	0.9	-	-
LS (Mehanee^13^)	3	16	-	1	-	0.0143

Significant values are in [bold].

Table 7 presents the search space and the inversion results from EKI alongside the comparative estimates from previously published techniques, including the DL Rao algorithm, BA, VFSA, R-imaging, and least squares (LS). EKI successfully recovered the model parameters with relatively tight uncertainty bounds, especially for depth (15.10 ± 1.1 m), horizontal position (–1.4 ± 0.05 m), and amplitude coefficient (4.06 ± 0.61 mGal·m²). These estimates are consistent with those produced by DL Rao and VFSA methods, while offering more interpretable and geologically plausible values than R-imaging or LS. The shape parameters (*q* = 1.00 ± 0.08 and *µ* = 0.90 ± 0.22) fall within the expected ranges for chromite bodies identified as horizontal cylinders. Drilling data provided additional support for the EKI inversion, indicating that the top of the chromite orebody lies at approximately 9.15 m, as shown in^3,6^. The EKI-derived model placed the anomaly centroid at 15.10 ± 1.1 m, which is consistent with the expected depth of a symmetric body and in agreement with physical observations. This correspondence highlights the method’s ability to deliver accurate and interpretable subsurface models.

**Fig. 15:**
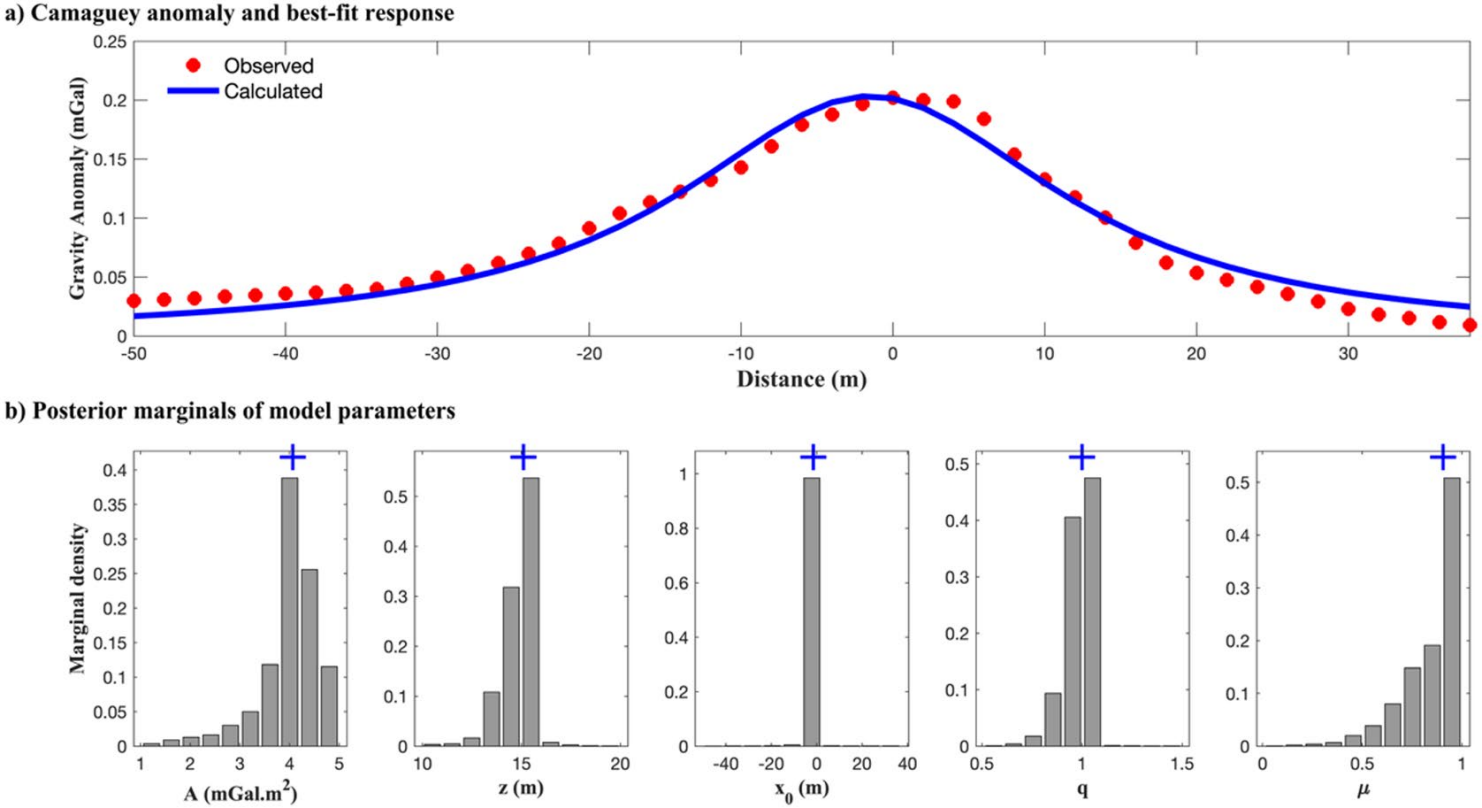
Inversion result of the Camaguey gravity anomaly using the regularized EKI: (a) the best-fit gravity response (blue line) shows strong agreement with the observed data (red points); (b) the marginal posterior distribution of the estimated model parameters, illustrating the uncertainty, concentration of ensemble solutions, and estimated value (blue ‘+’).

Figure 15a shows the gravity response computed from the best-fitting model, which closely matches the observed data with an RMSE of 0.0105 mGal. Compared with other methods (Table 7), EKI achieves one of the lowest RMSE values, indicating an excellent fit to the data. This value is comparable to that of the best-performing metaheuristic algorithms (DL Rao and VFSA) and significantly better than those of the remaining methods. Figure 15b shows the marginal posterior distributions of each parameter. The histograms are concentrated around the estimated values, particularly for depth and horizontal position. Such behaviour reflects high parameter identifiability and low inversion ambiguity. In contrast to traditional and metaheuristic methods, EKI provides point estimates and quantifies uncertainty across the ensemble, thereby enabling the probabilistic assessment of model reliability.

The Tikhonov regularization incorporated into EKI is pivotal for ensuring stable, and smooth updates, particularly for weakly sensitive shape factors (*q*, *µ*), which are otherwise prone to oscillation or overfitting. The regularized EKI algorithm successfully recovered geologically consistent subsurface structures from gravity data. The ability of EKI to produce reliable and uncertainty-informed estimates makes it a valuable tool for reducing exploration risk and guiding resource assessment.

### Faro anomaly Pb-Zn deposit, Yukon Territory, Canada

**Fig. 16:**
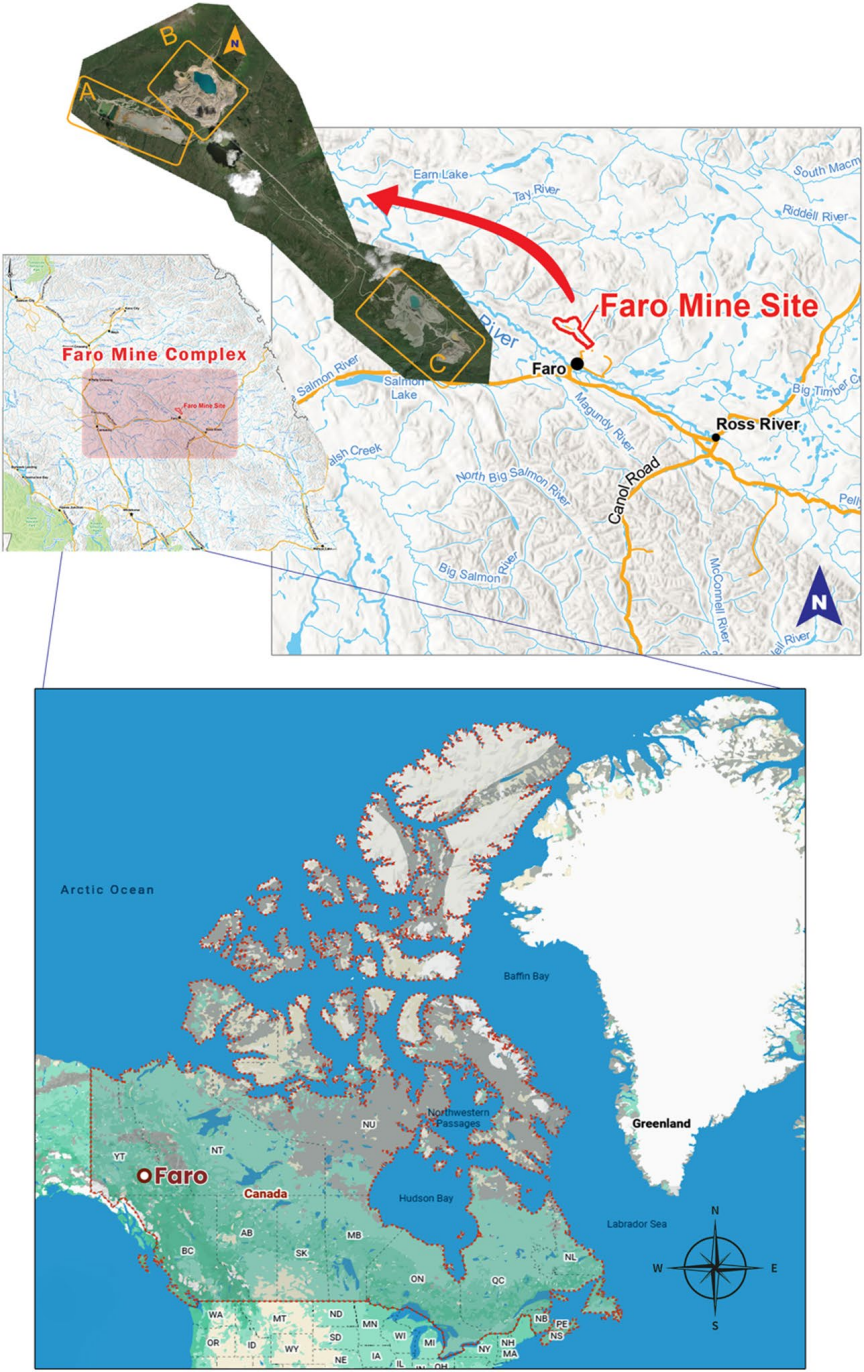
Site layout and location map of the Faro Mining District of Yukon, Canada (redrawn and modified after Tang^71^, Yukon Government^72^ using Adobe Illustrator CC 2024 (https://www.its.ac.id/dptsi/en/licensed-software/) under institutional license from Institut Teknologi Sepuluh Nopember).

**Fig. 17:**
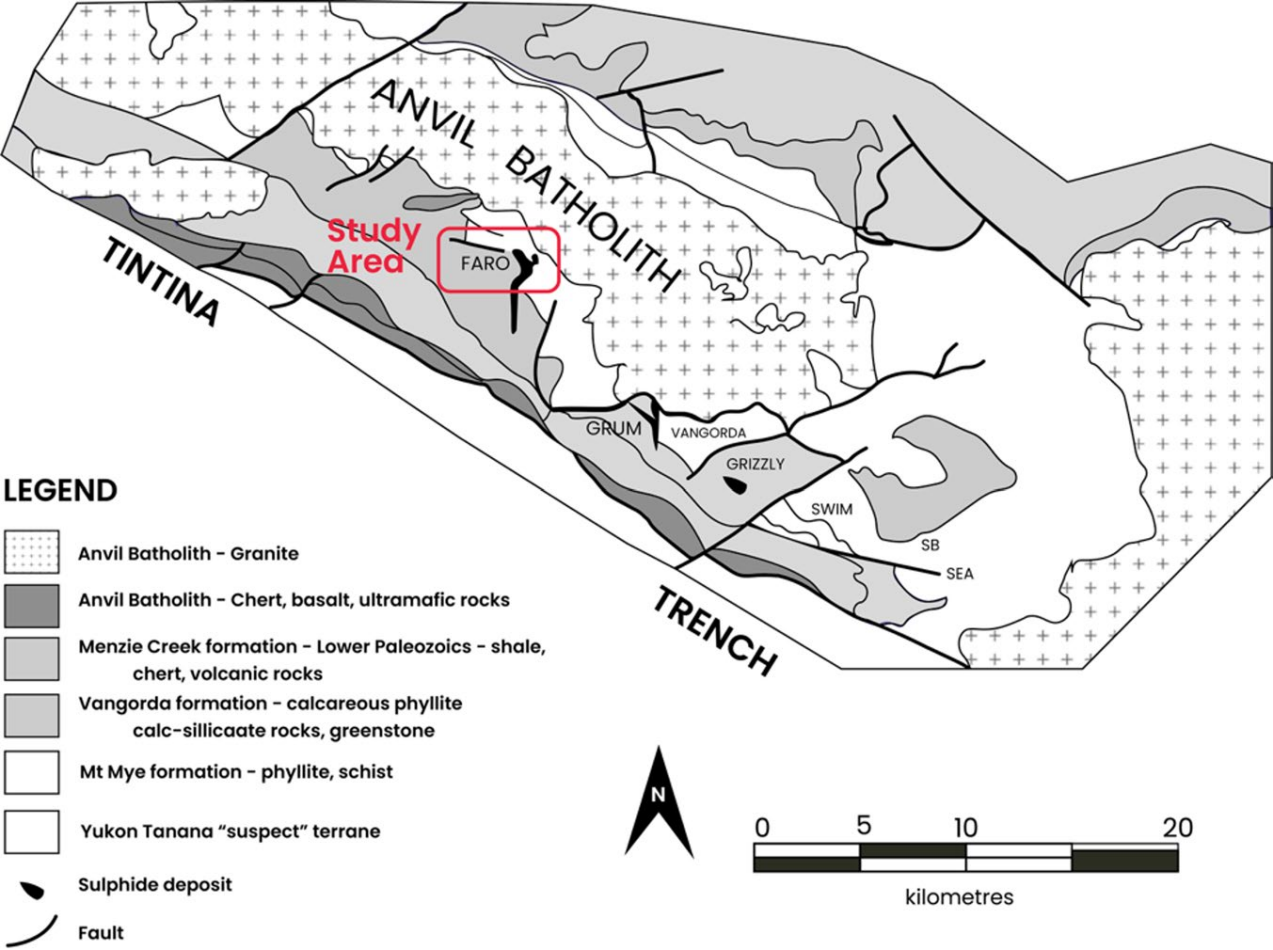
Geological map of the Anvil District, Canada, highlighting the location of the Faro sulphide ore deposit (redrawn and modified after Tang^71^ using Adobe Illustrator CC 2024 (https://www.its.ac.id/dptsi/en/licensed-software/) under institutional license from Institut Teknologi Sepuluh Nopember).

The Faro deposit is a major lead-zinc (Pb-Zn) orebody located in the Faro Mining District of Yukon, Canada (Fig. 16). This region has a longstanding history of mineral exploration, with gravity surveys playing a central role in delineating high-density mineralized zones as depicted in Fig. 17. Significant density contrasts typically characterize the Pb-Zn deposits in this area relative to the surrounding host rocks. For this study, a residual gravity anomaly profile approximately 230 m in length was collected perpendicular to the strike of the mineral deposit, enabling high-resolution subsurface modeling.

**Table 8:** Search space and inversion results for the Faro gravity anomaly using the regularized EKI compared with other methods from previously published studies.

Model parameter	${\text{A}}~\left( {{\text{~mGal}}.{{\text{m}}^2}{\text{~}}} \right)$	z (m)	D (m)	q	${\text{\varvec{\upmu}}}$	RMSE (mGal)
Search space	1–5	10–20	-50–38	0.5–1.5	0–1	
**EKI**	**546.17 ± 19.97**	**170.84 ± 4.47**	**-33.17 ± 0.96**	**1.00 ± 0.00**	**0.99 ± 0.01**	**0.0858**
DL Rao (Warnana et al.^5^)	546.97 ± 50.17	167.46 ± 1.58	-33.15 ± 0.08	1.01 ± 0.02	1 ± 0.02	0.0858
BA (Essa and Diab^3^)	490 ± 66.33	190 ± 25.81	-35 ± 1.39	1 ± 0.00	1 ± 0.01	0.1700
R imaging (Essa et al.^8^)	78.6	195	371.9	0.8	-	0.3300

Significant values are in [bold].

Table 8 presents the inversion results obtained using the regularized Ensemble Kalman Inversion (EKI) algorithm and the comparative solutions from previous studies, including the DL Rao algorithm, Bat Algorithm (BA), and R-imaging. The EKI method successfully estimated all model parameters with narrow uncertainty bounds, particularly for amplitude (546.17 ± 19.97 mGal·m^2^), depth (170.84 ± 4.47 m), and horizontal location (–33.17 ± 0.96 m). These values strongly agree with those reported by the DL Rao and BA methods, which demonstrated high gravity inversion performance. Notably, the estimated shape factor parameters (*q* = 1.00 ± 0.00, *µ* = 0.99 ± 0.01) are consistent with a horizontal cylinder-like body, a geometry commonly attributed to Pb-Zn deposits in sedimentary environments. These results also align with the borehole observations that placed the top of the ore body at a depth of approximately 76 m^3,5^, confirming the geologic plausibility of the EKI-derived model.

**Fig. 18:**
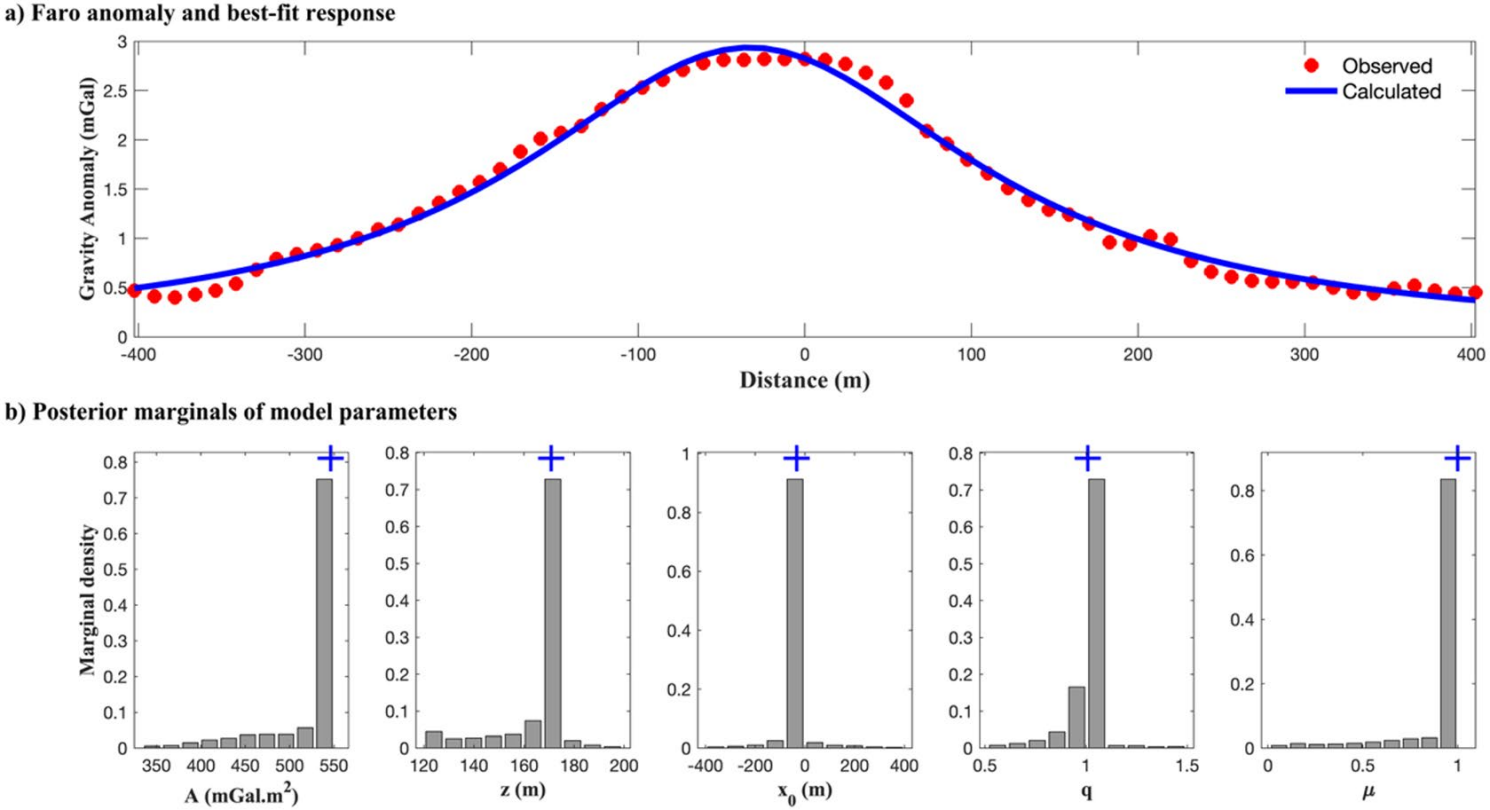
Inversion result of the Faro gravity anomaly using the regularized EKI: (a) the best-fit gravity response (blue line) shows strong agreement with the observed data (red points); (b) the marginal posterior distribution of the estimated model parameters, illustrating the uncertainty, concentration of ensemble solutions, and estimated value (blue ‘+’).

Figure 18a shows the best-fit gravity response generated by the EKI inversion. The model curve closely replicates the observed gravity data with an RMSE of 0.0858 mGal. This low residual error is comparable to that obtained with the DL Rao method and is substantially lower than that of the BA and R-imaging approaches (Table 8). Figure 18b shows the marginal posterior distributions of the inverted parameters. Most histograms were unimodal and sharply peaked around the median estimates, particularly for depth and lateral position. The narrow interquartile ranges across all parameters indicate strong ensemble agreement and low ambiguity in the recovered model, despite the challenge of imaging deep-seated ore bodies.

Generally, the regularized EKI demonstrates high accuracy, stability, and efficiency in modeling complex subsurface anomalies, such as the Faro Pb-Zn deposit. The integration of Tikhonov regularization enhances the numerical stability by improving the conditioning of the Kalman gain computation, particularly in the presence of noise or ensemble artifacts. Compared to previously published inversion techniques, EKI competes in accuracy and offers the added advantage of quantifying parameter uncertainties, making it a robust and interpretable tool for mineral exploration.

### Mobrun sulphide orebody, Canada

The Mobrun sulphide orebody is situated in a volcanogenic massive sulphide (VMS) environment in Rouyn-Noranda, Canada, where mineralization is hosted within volcanic and intrusive rock formations^74^. This area contains a significant concentration of cherty pyrite-dominated sulphide mineralization, accompanied by minor quantities of chalcopyrite, copper, zinc, and silver^73^. Gravity surveys conducted over the site revealed a distinct residual anomaly associated with the high density of the sulphide body. The gravity profile AB^7^, spanning 230 m with a 2-meter spacing, was used as input for inversion to recover the source parameters and delineate the subsurface geometry.

**Table 9:** Search space and inversion results for the mobrun gravity anomaly using the regularized EKI method compared to other methods in previously published studies.

Model parameter	${\text{A}}~\left( {{\text{~mGal}}.{{\text{m}}^2}{\text{~}}} \right)$	z (m)	D (m)	q	${\text{\varvec{\upmu}}}$	RMSE (mGal)
Search space	1–5	10–20	-50–38	0.5–1.5	0–1	
**EKI**	**58.65 ± 3.67**	**36.21 ± 0.18**	**-0.12 ± 0.02**	**0.76 ± 0.0**	**0.55 ± 0.02**	**0.0285**
DL Rao (Warnana et al.^5^)	96.66 ± 2.32	36.17 ± 0.52	-0.12 ± 0.0	0.77 ± 0.0	0.41 ± 0.01	0.0285
BA (Essa and Diab^3^)	80 ± 10.0	48 ± 6.83	0 ± 0.66	1 ± 0.0	1 ± 0.01	0.050
VFSA (Biswas^16^)	79.5 ± 0.7	47.7 ± 0.6	2.5 ± 0.4	1.0	-	0.060
R imaging (Essa et al.^8^)	38.47	47	2	0.91	1	-
LS (Mehanee^13^)	80	47	-	1	-	-

Significant values are in [bold].

Table 9 presents the search spaces and inversion results for the regularized EKI, along with comparative estimates from DL Rao, BA, VFSA, R-imaging, and LS methods. EKI achieved consistent recovery of all model parameters with relatively narrow uncertainty bounds, particularly for the amplitude coefficient (58.65 ± 3.67 mGal·m^2^), depth (36.21 ± 0.18 m), and location (–0.12 ± 0.02 m). These results closely align with the estimates obtained using DL Rao and VFSA methods, both of which are known for their robustness in handling complex inverse tasks. The shape factors (*q* = 0.76 ± 0.00, *µ* = 0.55 ± 0.02) suggest a source geometry consistent with a non-spherical, possibly horizontal cylinder, which aligns with the expectations for elongated sulphide lenses commonly observed in VMS-type deposits. In contrast, the LS and R-imaging methods produced broader or less physically plausible estimates, highlighting EKI’s ability to yield geologically realistic and statistically stable results. Notably, the estimated center of the orebody lies at 36.21 ± 0.18 m, while drilling reports place the top of the sulfide mineralization at approximately 23.34 m depth^3,5^, indicating that the inversion depth is consistent with the expected geometry of the deposit.

**Fig. 19:**
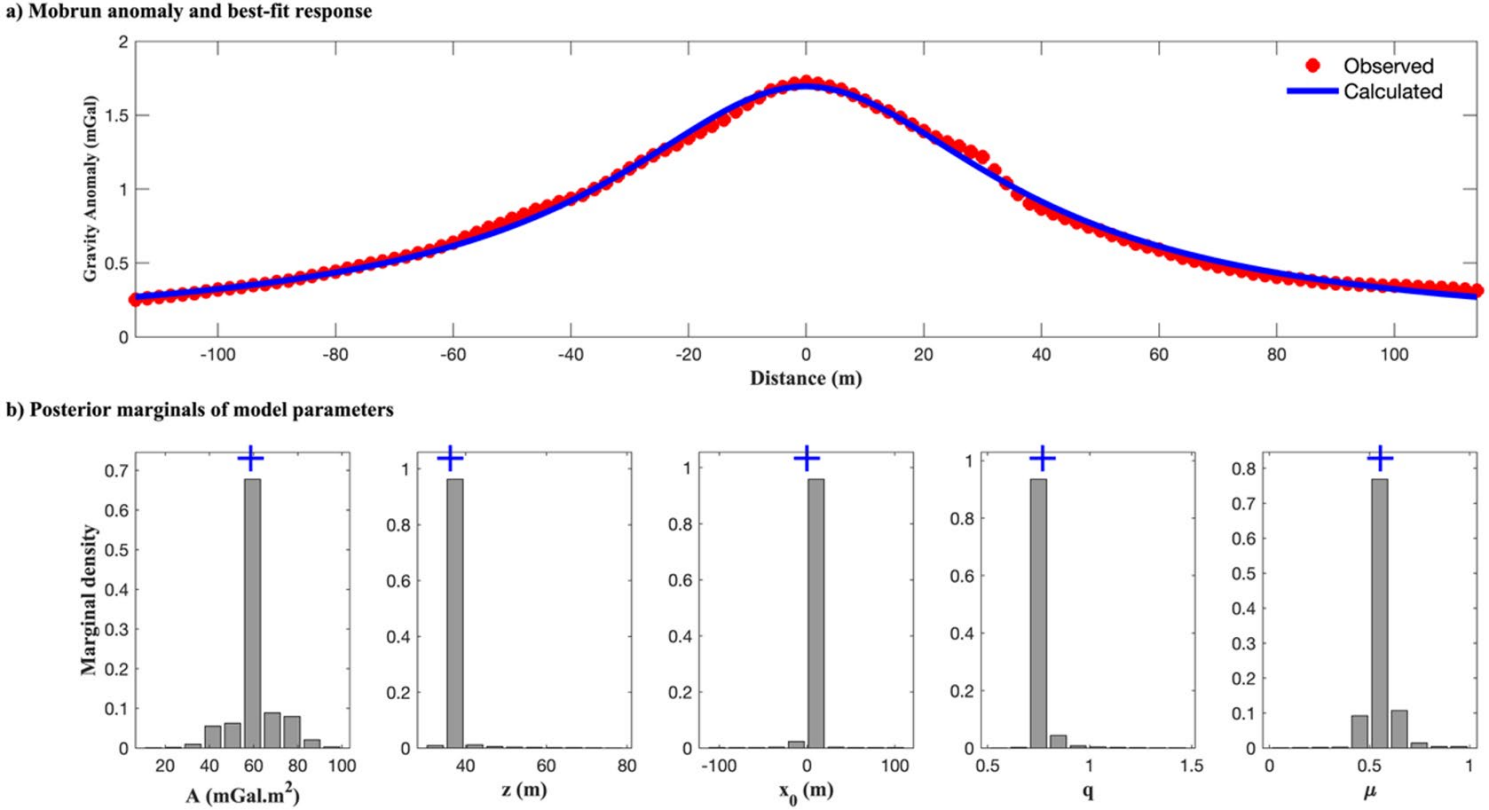
Inversion result of the Mobrun gravity anomaly using the regularized EKI: (a) the best-fit gravity response (blue line) shows strong agreement with the observed data (red points); (b) the marginal posterior distribution of the estimated model parameters, illustrating the uncertainty, concentration of ensemble solutions, and estimated value (blue ‘+’).

Figure 19a shows the gravity response computed from the EKI-derived best-fit model. The calculated gravity closely fits the observed data, yielding an RMSE of 0.0285 mGal, which is, identical to that obtained by DL Rao (Table 9). Compared with VFSA and BA, EKI demonstrates superior data-fitting performance. The marginal posterior distributions (Fig. 19b) are sharply peaked and centered near the estimated values for key parameters, particularly depth and location. These narrow distributions indicate a reduced ensemble spread and strong parameter identifiability. Moreover, the consistently small interquartile ranges across all parameters highlight high ensemble consistency and low model ambiguity, even when imaging deep-seated ore bodies.

Applying the regularized EKI to the Mobrun dataset demonstrates its robustness in recovering reliable parameters in a moderately complex geological setting. Tikhonov regularization enhances the numerical stability of the inversion, particularly when resolving shape parameters (*q* and *µ*), which are often poorly constrained in ill-posed gravity data inversion. Compared to metaheuristic methods, which often require extensive parameter tuning, EKI provides an efficient and interpretable inversion workflow. The ability to quantify uncertainty alongside parameter estimation makes it a valuable tool for early-stage exploration and resource targeting in sulfide-rich terrains.

### Mundiyawas Cu-Au deposit, India

**Fig. 20:**
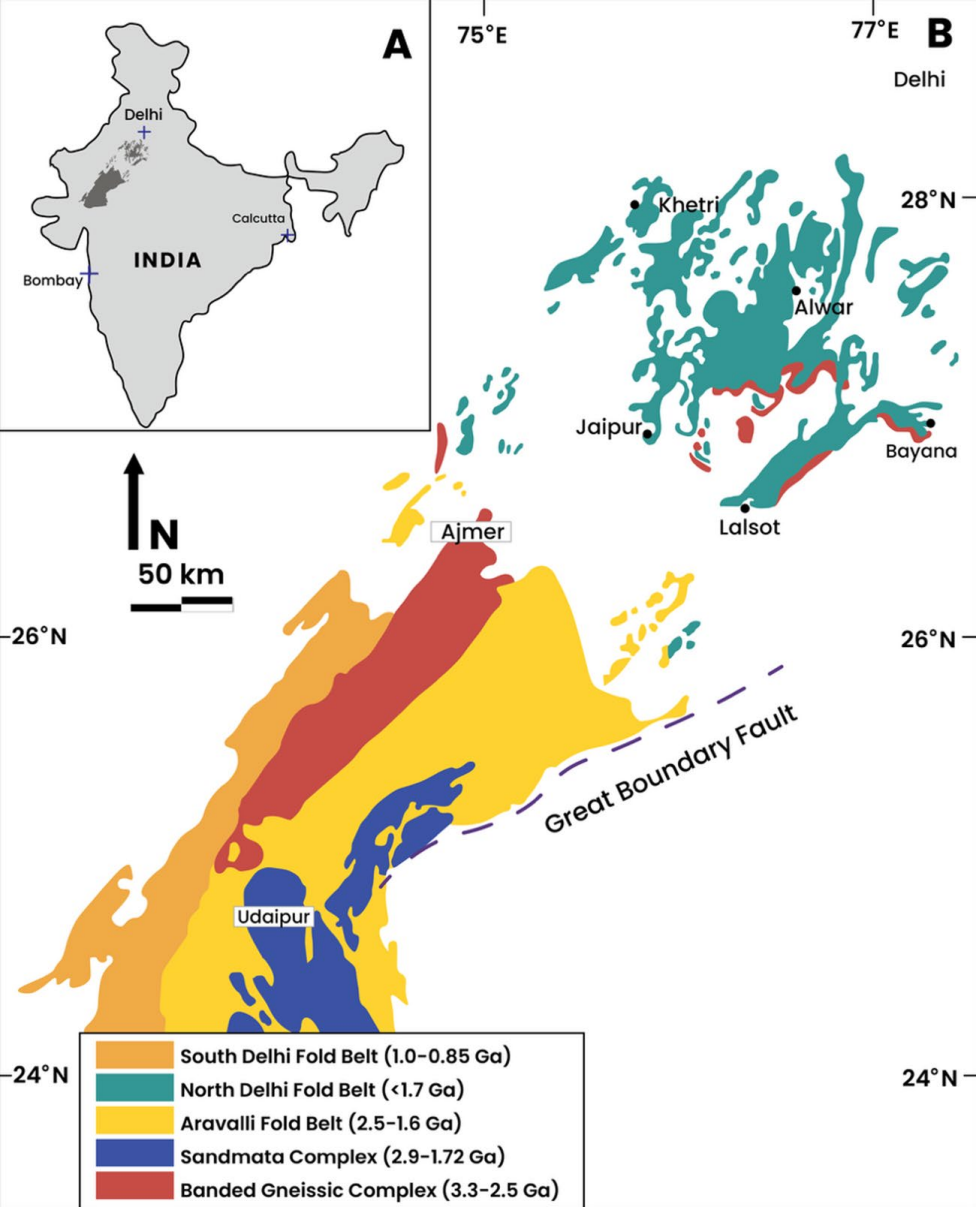
Location of the Alwar Basin within the North Delhi Fold Belt (NDFB), India, where the Mundiyawas area is situated (redrawn and modified after Sahoo et al.^75^ using Adobe Illustrator CC 2024 (https://www.its.ac.id/dptsi/en/licensed-software/) under institutional license from Institut Teknologi Sepuluh Nopember).

**Fig. 21:**
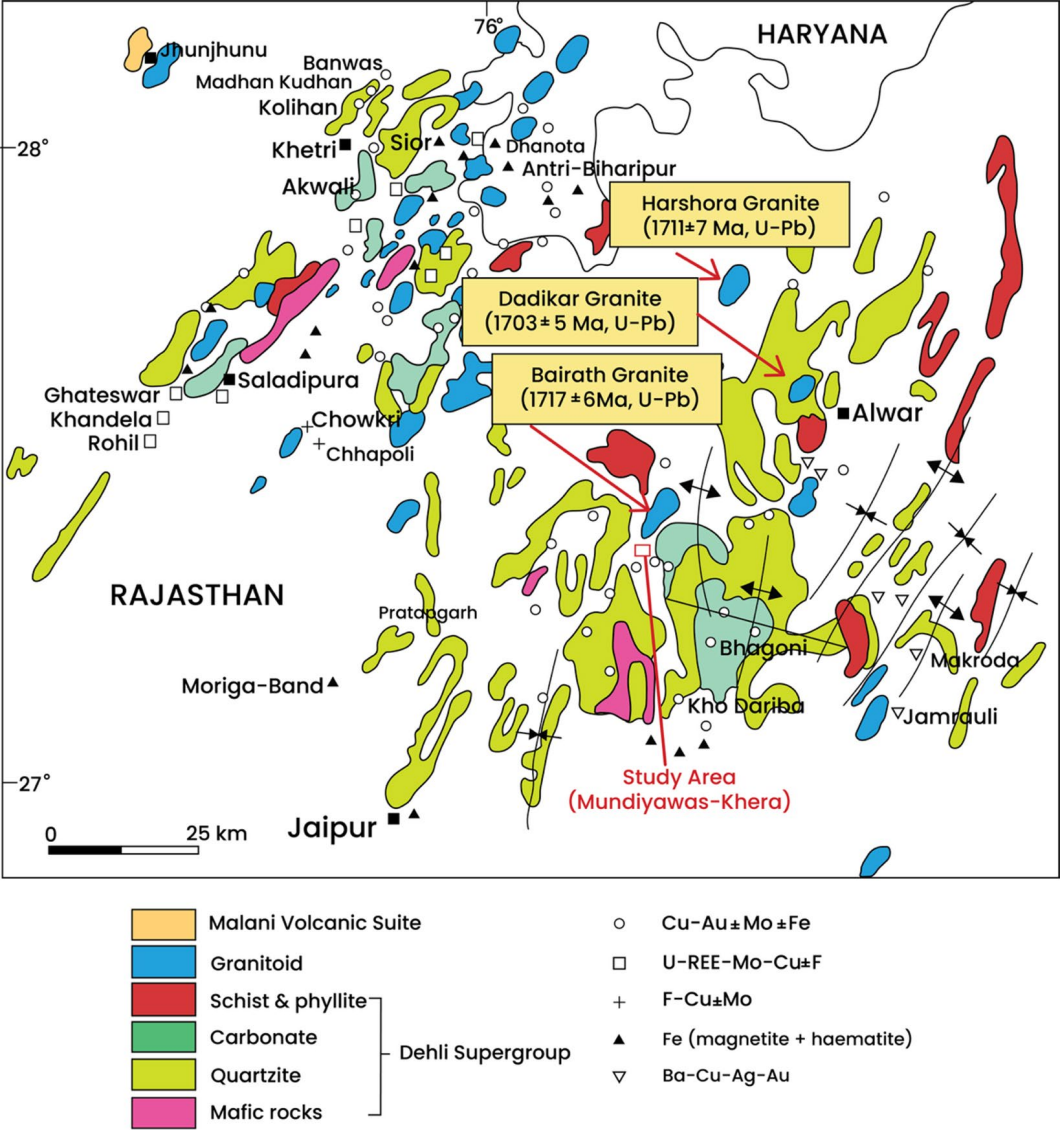
Geological map of the Alwar basin displaying our study area, the Mundiyawas-Khera region (redrawn and modified after Sahoo et al.^75^ using Adobe Illustrator CC 2024 (https://www.its.ac.id/dptsi/en/licensed-software/) under institutional license from Institut Teknologi Sepuluh Nopember).

The Mundiyawas-Khera region, located within the Alwar Basin in the North Delhi Fold Belt (NDFB), India, is known for its copper (Cu) and gold (Au) mineralization, which is primarily hosted within Proterozoic felsic metavolcanic rocks (Figs. 20 and 21). The broader NDFB is a prominent metallogenic zone within the northwestern Indian plate, comprising three major volcano-sedimentary basins: Bayana, Alwar, and Khetri^4,75^. The Mundiyawas area is further characterized by tremolite-bearing dolomites, carbonaceous phyllites, and mica schists^75^, all of which are overlain by soil and scree layers of variable thickness (1.5–3 m). Airborne gravity surveys in this region have revealed complex, overlapping anomalies interpreted as multiple buried ore bodies at varying depths.

**Table 10:** Search space and inversion results for the Mundiyawas Cu–Au gravity anomaly with regularized EKI compared to previously published results.

Anomaly	Model parameter	Search space	EKI	DL Rao (Warnana et al.^5^)	BA (Essa and Diab^3^)
1) Deep body	${A_1}\left( {{\text{~mGal}}.{{\text{m}}^2}{\text{~}}} \right)$	10–500	**498.99 ± 4.05**	415.34 ± 95.01	245 ± 2.5
	${z_1}\left( m \right)$	1–200	**114.50 ± 0.78**	110.45 ± 22.99	110 ± 5.0
	${x_{01}}\left( m \right)$	-640–640	**-212.26 ± 0.73**	-210.78 ± 2.18	-205 ± 18.08
	${q_1}$	0.5–1.5	**1.06 ± 0.0**	1.05 ± 0.06	1 ± 0.4
	${\mu _1}$	0–1	**0.99 ± 0.0**	1 ± 0.14	1 ± 0.01
2) Shallow body	${A_2}\left( {{\text{~mGal}}.{{\text{m}}^2}{\text{~}}} \right)$	10–500	**499.08 ± 17.31**	482.11 ± 107.22	273 ± 1.5
	${z_2}\left( m \right)$	1–200	**106.92 ± 0.76**	104.49 ± 11.79	90 ± 3.33
	${x_{02}}\left( m \right)$	-640–640	**52.99 ± 0.25**	52.76 ± 1.43	50 ± 4.73
	${q_1}$	0.5–1.5	**1.04 ± 0.01**	1.04 ± 0.22	1 ± 0.4
	${\mu _1}$	0–1	**0.99 ± 0.01**	0.98 ± 0.43	1 ± 0.4
RMSE (mGal)			**0.0704**	0.0691	0.1300

Significant values are in [bold].

The residual gravity profile AA’ analyzed in this study spans 1,280 m and was digitized at 10-meter intervals, as shown in^4^. The profile features of the two major gravity highs were suspected to represent deep and shallow mineralized sources. The inversion was performed using a dual-anomaly model within the regularized EKI framework. The results are summarized in Table 7, along with comparative estimates from the DL Rao and Bat Algorithm (BA) methods.

The regularized EKI algorithm effectively resolved both high-fidelity anomalies and well-constrained uncertainty bounds. The deeper body was estimated to have an amplitude of ${A_1}$= 498.99 ± 4.05 mGal·m^2^ and a center depth of ${z_1}$​= 114.50 ± 0.78 m, consistent with prior interpretations and drilling evidence of buried sulfide-bearing felsic volcanics (Rao et al., 2019). The shallow body was delineated with a similar amplitude of ${A_2}$​= 499.08 ± 17.31 mGal·m^2^ and center depth of ${z_2}$​= 106.92 ± 0.76 m. Notably, the estimated horizontal locations of both bodies (${x_{01}}$​= −212.26 ± 0.73 m and ${x_{02}}$​= 52.99 ± 0.25 m) correspond closely with the mapped anomaly peaks, supporting the structural interpretation of the profile.

**Fig. 22:**
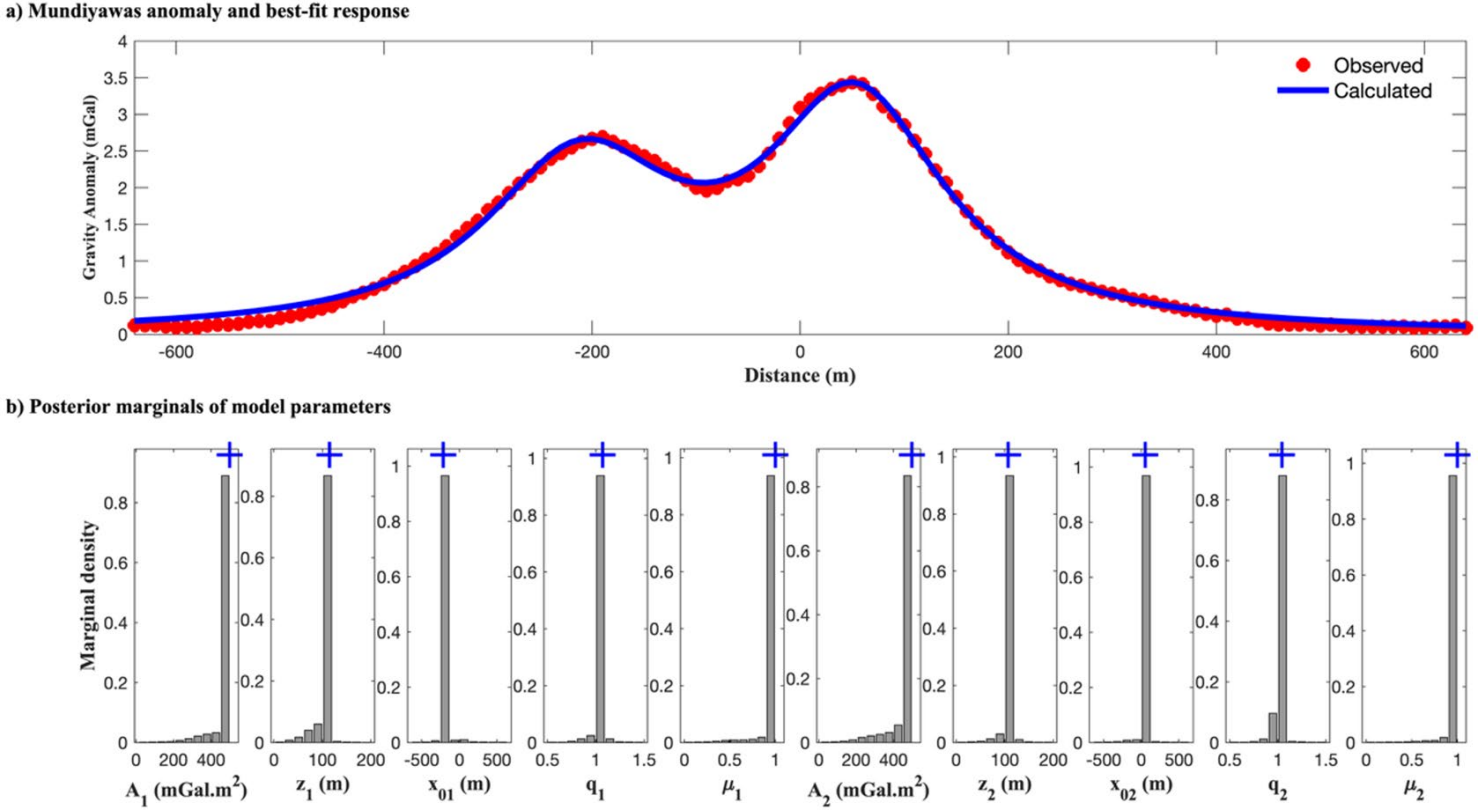
The search space and the inversion result of the Mundiyawas Cu–Au gravity anomaly using regularized Ensemble Kalman Inversion (EKI): (a) Comparison between the observed residual gravity anomaly and the gravity response computed from the best-fit EKI model; (b) Marginal posterior distributions of the model parameters for both deep and shallow anomalies, illustrating the stability and ensemble consistency of the inversion.

Figure 22a presents the gravity response calculated from the EKI best-fit model, which closely fits the observed residual gravity profile. The resulting RMSE of 0.0704 is comparable to that of DL Rao and clearly outperforms BA (Table 10). Figure 22b shows the posterior distributions of each parameter. While the amplitude and depth parameters exhibit narrow, sharply peaked distributions indicative of strong identifiability, the shape factors (*q* and *µ)* show broader but still physically consistent spreads, reflecting the inherent difficulty in isolating geometry under overlapping conditions.

The EKI framework demonstrated superior performance in resolving the composite anomaly structure compared to published results. Its solutions are both geologically plausible and statistically reliable, with tighter uncertainty bounds than those produced by the BA and DL Rao methods. Tikhonov regularization is key in suppressing instability, particularly in the shape parameters. It allows the inversion to remain stable and interpretable even under challenging structural overlap. In summary, the Mundiyawas inversion underscores the capacity of the regularized EKI framework to address complex, high-dimensional gravity inversion problems in real mineralized terrains. Its ability to produce stable, interpretable, and uncertainty-aware solutions makes it a powerful tool for geophysical exploration in structurally intricate settings.

## Conclusions

This study developed a regularized ensemble Kalman inversion (EKI) for robust and efficient gravity data modelling to identify mineral and ore deposits. By incorporating Tikhonov regularization $\left( {\lambda I} \right)$ into the Kalman gain computation, the method effectively mitigates the numerical instabilities arising from the ill-conditioned covariance matrix of the Kalman gain, particularly under low ensemble variability or noisy data conditions. An ensemble size experiment demonstrated that using ${N_e} \geqslant 100$ improved the estimates and reduced posterior spread. Sensitivity analysis indicated that moderate regularization $\left( {1e - 3 \leqslant \lambda \leqslant 1e - 1} \right)$ provides an optimal balance between stability and resolution, with $\lambda$ functions as a numerical safeguard in the Kalman gain computation. Systematic benchmarking against established metaheuristic algorithms (PSO, VFSA, and BA) showed that the regularized EKI converges faster and exhibits superior stability while maintaining comparable accuracy, demonstrating its robustness and efficiency. Synthetic and real gravity data in mineral exploration (including chromite, Pb-Zn, sulphide, and Cu-Au deposits) demonstrated reliable, geologically consistent results that align with drilling information. Overall, the proposed framework advances ensemble-based inversion toward automated, uncertainty-aware exploration workflows. Future work will focus on adaptive regularization to enhance flexibility and data-driven implementation across diverse geophysical problems.

## Data Availability

The data set analyzed in this study is available upon request from the corresponding author Dharma Arung Laby (email: dharma.arunglaby@its.ac.id).
